# Insights of semantic segmentation using the DeepLab architecture for autonomous driving

**DOI:** 10.1016/j.mex.2025.103387

**Published:** 2025-05-23

**Authors:** Javed Subhedar, Mrinal R Bachute

**Affiliations:** Department of Electronics and Telecommunication Symbiosis Institute of Technology, Pune, India

**Keywords:** Autonomous driving, CBAM, Semantic segmentation, DeepLab, Semantic Segmentation Using the DeepLab Architecture for Autonomous Driving

## Abstract

One of the critical tasks of autonomous driving systems is the Perception task (detecting the surroundings), which involves semantic Segmentation. The vital computer vision task of semantic segmentation assigns a “label” to every pixel in the input image. “Semantic segmentation” task consists of partitioning scenes as seen by the Autonomous Vehicle into several communicative slices by categorizing and labelling all image pixel for semantics. This paper gives insights into DeepNet V3 + architecture with ResNet50V2 as the backbone and the other as EfficientNetv2 backbone for feature extraction. The impact of the Squeeze and Excitation module and the Convolutional Block Attention Module is also compared for these architectures for semantic segmentation using the CAMVid data set. All six models are evaluated for Categorical Accuracy and mIoU metrics. The maximum Categorical Accuracy of 97.25 % was achieved in the model ResNet50V2 as the backbone and the Mean IoU of 80.56 %

• Feature extraction using DeepNet V3 + architecture with ResNet50V2 and EfficientNetv2 as the backbone.
• Insights of using the Squeeze and Excitation and Convolutional Block Attention Module for the DeepNet V3 + architecture.

## Graphical abstract

**Figure:**
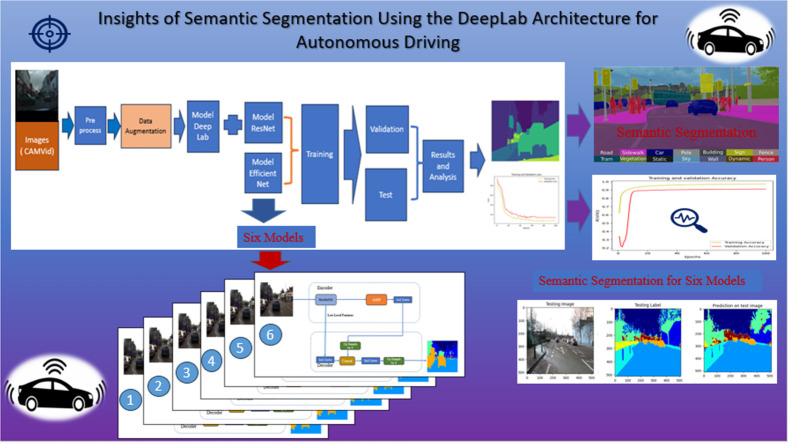

Specifications table

**Table:** 

Subject area:	Engineering
“More specific subject area”:	Autonomous Driving
Name of your method:	Semantic Segmentation Using the DeepLab Architecture for Autonomous Driving
“Name and reference of original method”:	Semantic Segmentation
Resource availability:	Li et al., “Lane-DeepLab: Lane semantic segmentation in automatic driving scenarios for high-definition maps, doi:10.1016/j.neucom.2021.08.105.”

## Background

### Autonomous driving systems

**Fig. 1:**
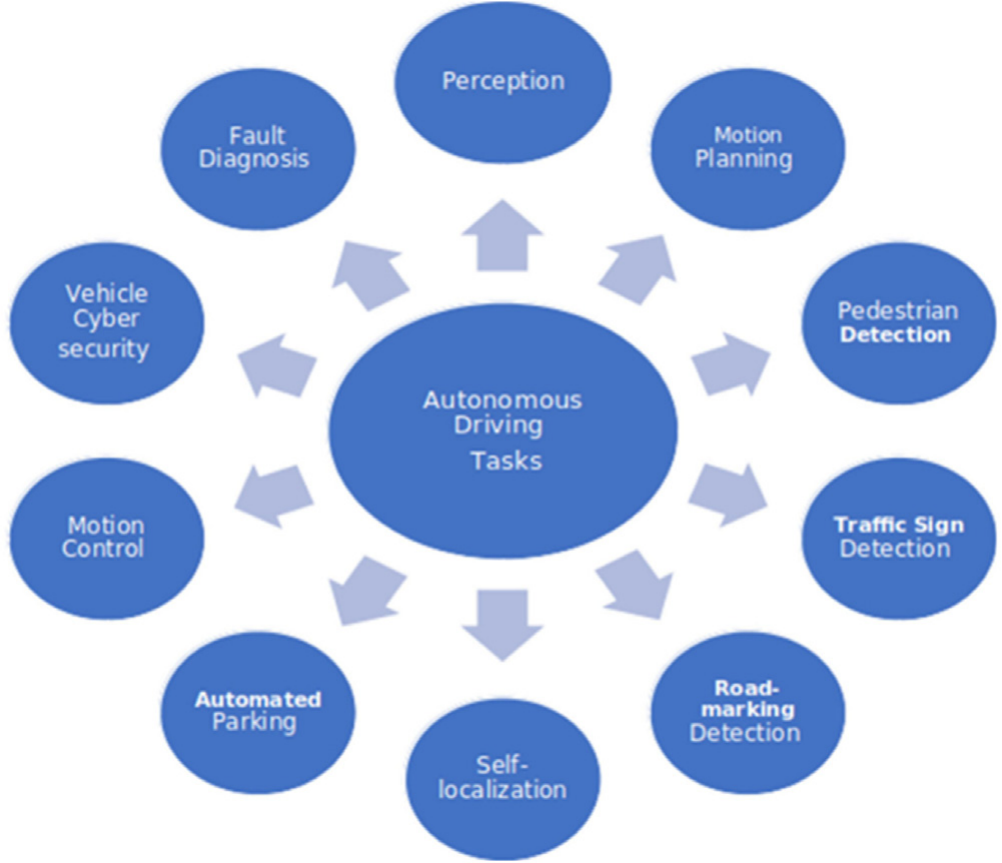
Autonomous driving system [2].

Autonomous Driving systems have the distinct advantage of eradicating the likelihood of human mistake or meagre human choices (e.g. commotion) while driving [1]. Thus, it has massive potential to save lives and decrease the fiscal burden linked with crashes. The potential financial and societal benefits of AVs could also be substantial, including increased economic productivity and efficiency, reduced commuting time, and even the possible reduction of the environmental impact of conventional surface vehicles while improving overall system energy efficiency. Autonomous driving provides mobility to citizens facing transportation challenges, increasing their access to jobs and services and their ability to live independently. Automated vehicles (AVs) promise a safer driving experience than conventional, human-driven vehicles. An Autonomous Driving system is a complex system. The different tasks [2] to be done in an Autonomous Driving system are shown in Fig. 1.The different tasks include Motion planning, Traffic signal detection, and Motion control, which in turn involves acceleration/deceleration, turning left/right, reversing, Fault diagnosis, etc. One of the critical tasks is the perception task, which involves perceiving the objects around the vehicle for taking further control action. This identification is done through semantic segmentation of the images captured by the camera sensors mounted on the Automated vehicle. There are many challenges for deep multi-modal object detection and semantic segmentation in autonomous driving [3].

**Fig. 2:**
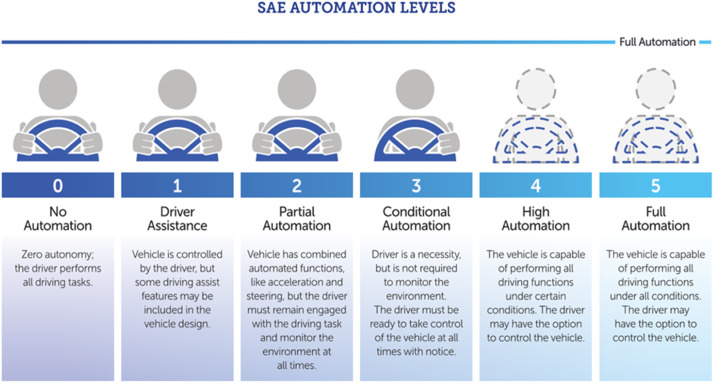
Autonomous driving systems levels [5].

The Autonomous driving systems are categorised under levels 0 (lowest) to 5 (highest) as per the SAE standard J3016 [4] Level 0 refers to No Driving Automation, and Level 5 refers to Full Driving Automation. These levels are shown Fig. 2.

### Semantic segmentation

The successful implementation of deep convolution neural networks for image cataloguing has ignited curiosity in leveraging their potential for cracking pixel-based tasks like “Semantic Segmentation” which necessitates multi-scale circumstantial reasoning and dense, complete resolution estimates. “Semantic Segmentation” is partitioning a “digital image” into multiple segments (sets of pixels, also known as image objects). Semantic image segmentation predicts whether each pixel of an image is associated with a specific class [6]. Semantic image segmentation contrasts object detection, which detects objects in rectangular regions, and image classification, which classifies the overall image. Segmentation aims to simplify and change the representation of an image into something more meaningful and accessible to analyze. Different deep-learning models for semantic Segmentation aim to assign semantic labels (e.g., car, tree, pedestrian, etc.) to each pixel in the input image. In current years, deep learning has accomplished substantial achievement in “semantic segmentation” across diverse arenas. “Convolutional neural networks” can inevitably learn features from images and excerpt top semantic data to accomplish precise scene recognition.

In comparison with verge-based Segmentation and conventional machine learning techniques, an essential advantage of Convolution Neural Networks as they do not need verge choice or feature engineering. Feature and semantic data are mined inevitably from the information samples through “convolutional layers” throughout network training. Some of the deep learning models for the semantic Segmentation are:

#### Pyramid scene parsing network (PSPNet)

**Fig. 3:**
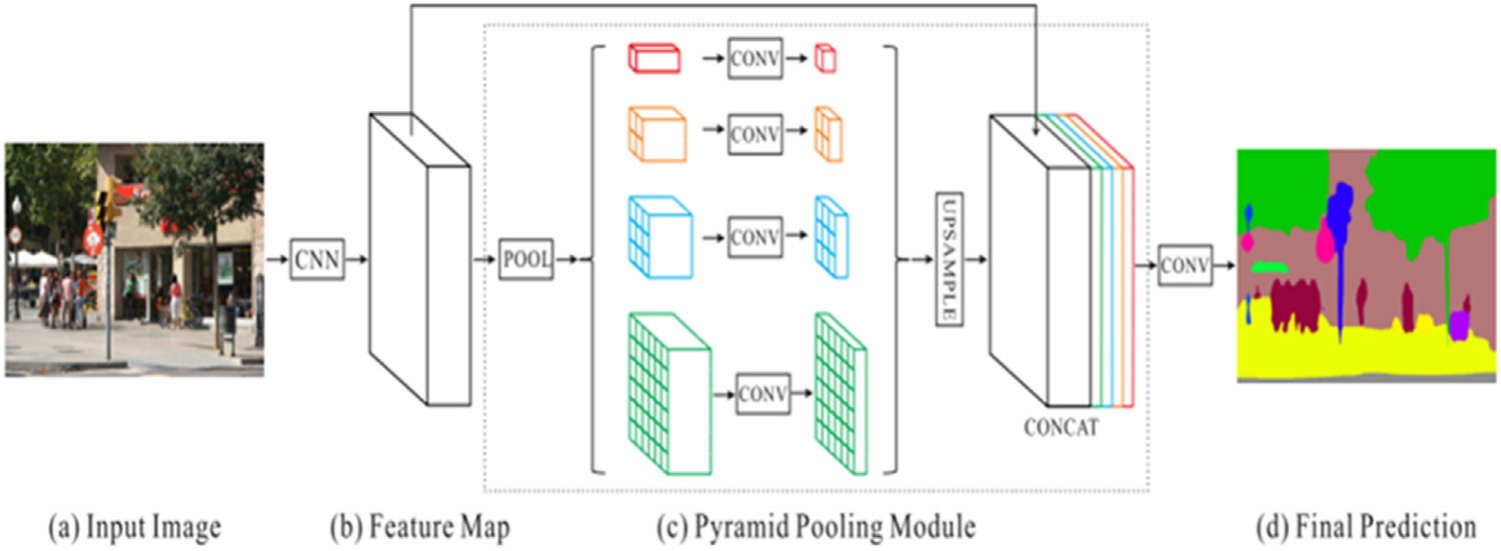
Pyramid scene parsing network (PSPNet) Architecture [7].

In the Pyramid scene parsing network [7] the pyramid parsing module is applied to the last convolution layer of the Convolution Neural Network (CNN). This module fuses the features of many pyramid scales to add local and global context data, fed into the “convolution layer” to achieve the final per-pixel prediction. The “Pyramid scene parsing network (PSPNet) Architecture” is shown in Fig. 3. The Fig 3(a) shows the input image for which the semantic segmentation needs to be done. The Fig 3(b) represents the feature map. The Fig 3(c) shows the Pyramid pooling module, which has convolution layers, and lastly, the Fig 3(d) gives the final prediction by the PSPNet network.

#### SegNet

**Fig. 4:**
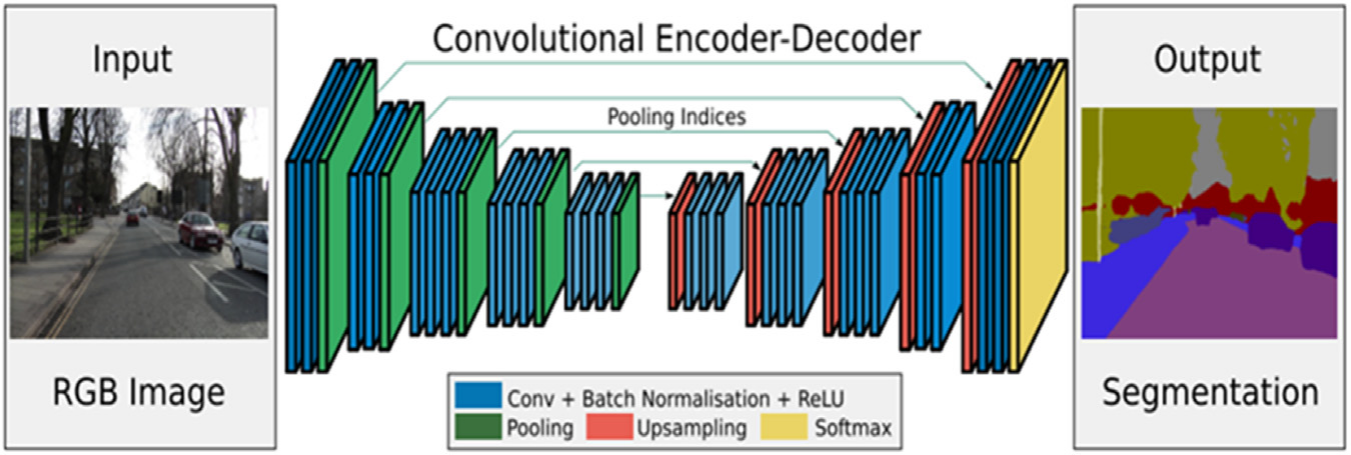
SegNet architecture [8].

The SegNet [8] is a convolution neural network with an “encoder-decoder network with skip connections”. In this network, each decoder maps the low-slung resolution features of an encoder layer to advanced resolution feature map. Implementation of up-sampling requires the decoder to use the “pooling indices” in the corresponding encoder’s “max-pooling” (down-sampling) computation. Thus, the need to learn the up-sampling is eliminated, resulting in fewer parameters. Sharper segmented boundaries are obtained by using this network. SegNet is a symmetric architecture, as the decoder is an exact mirror of the encoder. The SegNet architecture is shown in Fig. 4

#### UNET

The model consists of a contracting path on the left and an expansive one on the right [9]. The contracting path is a “convolutional network” of recurrent application of two 3×3 convolutions, each followed by a “Rectified Linear Unit (ReLU)” and a 2×2 “max pooling” act with stride 2 for “down-sampling”. The number of “feature channels” is doubled at every “down-sampling” step. Upsampling of the “feature map” is included in individual step in the “expansive path”. A 2×2 convolution follows that splits the numeral of feature channels, a “concatenation” with the “congruently cropped” feature map from the contracting path, and two 3×3 convolutions, each followed by a “ReLU”. UNET Model is shown in

**Fig. 5:**
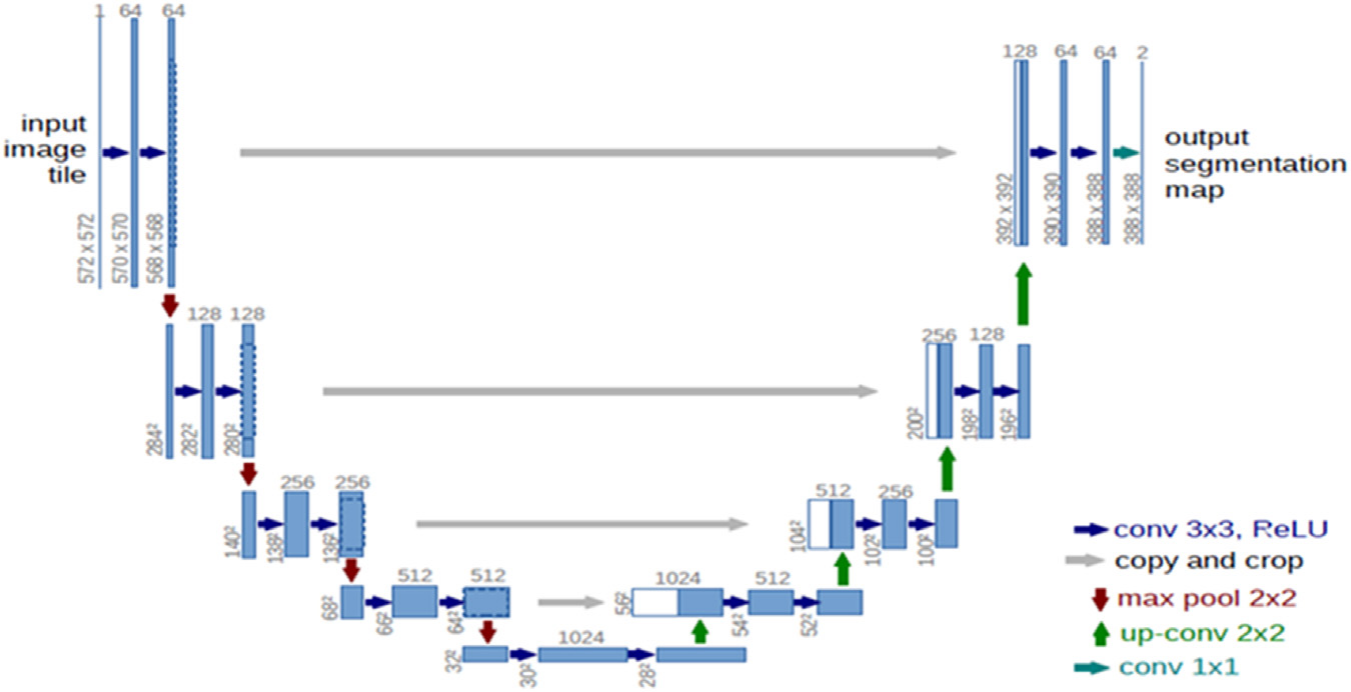
UNET architecture [9].

Fig. 5 and can be used for optimization of semantic segmentation [10].

### Related work

DeepLab is a advanced semantic segmentation model premeditated and open-sourced by Google. The dense estimate is attained by just “up-sampling” the output of the final convolution layer and calculating pixel-wise loss. The DeepLab smears atrous convolution for up-sample. DeepLab architecture combines two previously known methods: “Deep Convolutional Neural Networks” (DCNNs) and fully connected “Conditional Random Fields” (CRFs). DeepLab ­networks are “deep learning networks” open-sourced by the “Google research team”, which has introduced “Atrous ­Convolution”, “Conditional Random Field (CRF)”, and “Atrous Spatial Pyramid Pooling (ASPP)” modules in sequence. These modules completely utilise the feature graph’s multi-scale data, enhancing the model’s capability to arrest fine details and hovering the performance of the deep learning semantic segmentation network to next level. DeepLabv3+ augments a simple but “effective decoder module” based on DeepLabv3, improving the model’s ability to deal with image borders and better preserving the target’s edge. The resolution of “coding features” can be output using the anticipated “encoder-decoder” structure by monitoring the “atrous convolution”, and the accuracy and running time can be well-adjusted.

The three main advantages of the “DeepLab system” are

(i) Speediness by the ‘atrous’ algorithm
(ii) exactness
(iii) easiness, comprising a combine of two deep-rooted modules, “DCNNs” and “CRFs”.

The “DeepLab” system re-purposes networks trained on “image classification” to semantic Segmentation by smearing the ‘atrous convolution’ with sampled filters for “dense feature abstraction” . It is extended to atrous spatial pyramid pooling, which encodes objects and image context at multiple scales. The ideas from deep convolutional neural networks and fully connected conditional random fields are combined to produce semantically accurate predictions and detailed segmentation maps along object boundaries.

The DeepLab family has four architectures, namely the DeepLabv1, DeepLabv2, DeepLabv3, and DeepLabv3+. The first two versions use Deep Convolutional Neural Networks to extract feature maps, using atrous convolutions, followed by fully connected conditional random fields to smooth boundaries and recover more accurate maps. DeepLabv3 uses only DCNNs with atrous convolutions and spatial pooling operations to improve performances and make end-to-end learning possible [11]. Finally, the DeepLabv3+ has an encoder-decoder architecture to increase the performance of object boundary recovery and segmentation accuracy.

#### DeepLab V1

**Fig. 6:**
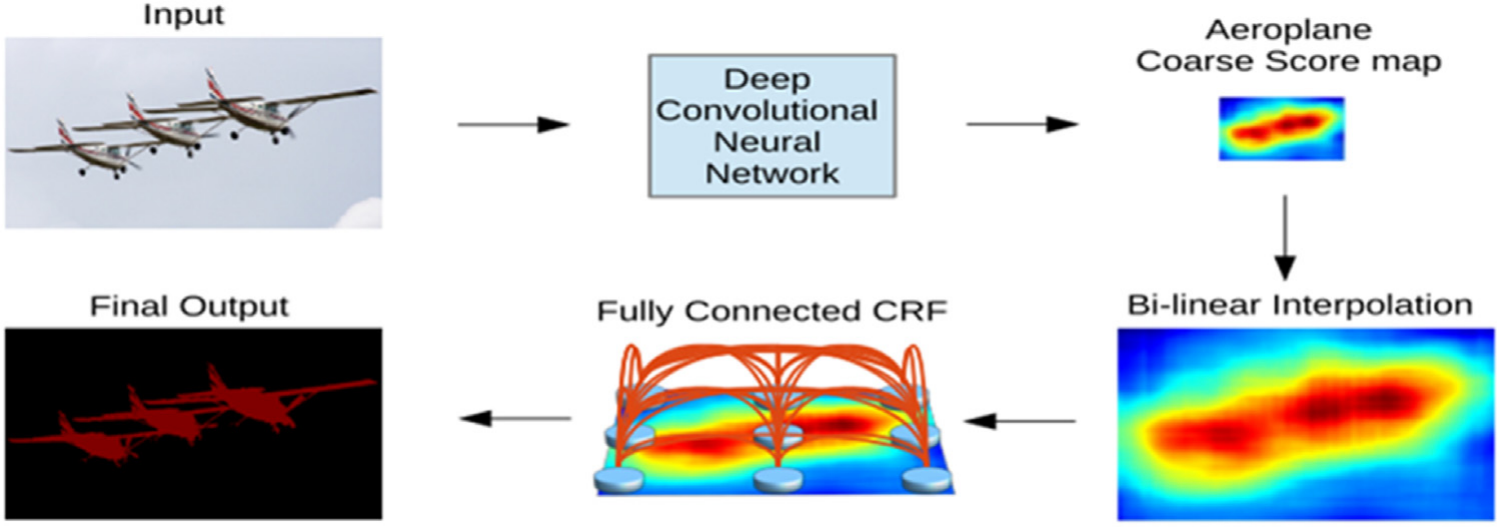
DeepLab V1 architecture [11].

DeepLab V1 is the first version of the DeepLab architecture. This version proposed a novel approach to semantic image segmentation that comprehended the combination of two previously known methods: “Deep Convolutional Neural Networks” (DCNNs) and fully connected Conditional Random Fields (CRFs). DeepLabv1 uses only VGGNet. The DeepLab V1 architecture is shown in Fig. 6

#### DeepLab V2

**Fig. 7:**
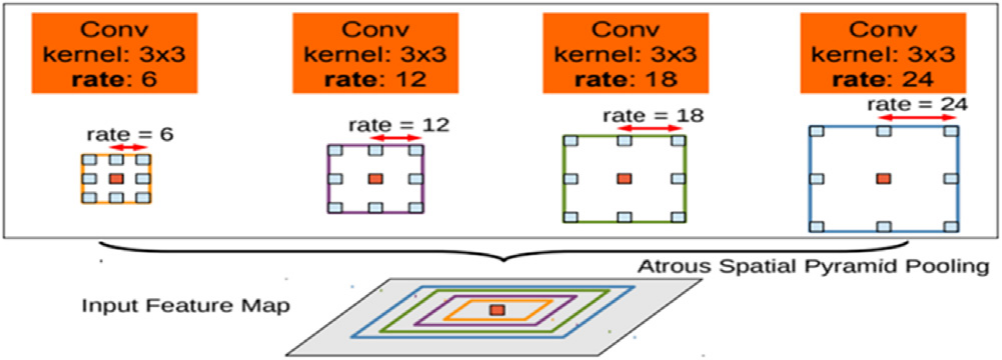
Atrous spatial pyramid pooling (ASPP) [11].

The second version of DeepLab, DeepLabv2 was presented in 2017 by the same authors. This version usages “Atrous Spatial Pyramid Pooling (ASPP)”, revealed in Fig. 7, to “vigorously segment” objects at various scales with filters at numerous sampling rates and effective fields-of-views as the changes from the previous version were not substantial, with the overall architecture remaining the same: a combined DCNN and CRFs with post-processing to smooth edges. The main addition is the usage of Atrous Spatial Pyramid Pooling (ASSP) in the convolutional layers. To categorize the center pixel, “ASPP” feats multi-scale features by engaging several parallel filters with dissimilar rates. The effective “Field-Of-Views” are shown in various colors. DeepLabv2 uses ResNet and VGGNet architecture. The “Atrous Spatial Pyramid Pooling module” has five branches: one 1 by 1 convolution, three 3 by 3 convolution with numerous “atrous rates”, and “pooled image feature”.

#### DeepLab V3

The third version of the DeepLab architecture was proposed in December 2017 [12] and aimed to improve the performance of the previous approaches. In this version, the “ASPP module” with the “image-level feature” captures longer-range data and includes “batch normalisation parameters” to facilitate the training. In particular, “atrous convolution” is applied to excerpt “output features” at dissimilar output strides while training and evaluation. This efficiently authorizes “training at an output stride” equivalent to 16 and attains a “high performance” at an “output stride” of eight during evaluation. The structure of “DeepLabv3” is similar to that of DeepLabv2, using DCNN followed by ASPP, but the final CRF procedure is removed from the network to allow for end-to-end learning. Despite removing the smoothening layer, the network’s performance surpasses the previous architectures.

#### DeepLab V3+

**Fig. 8:**
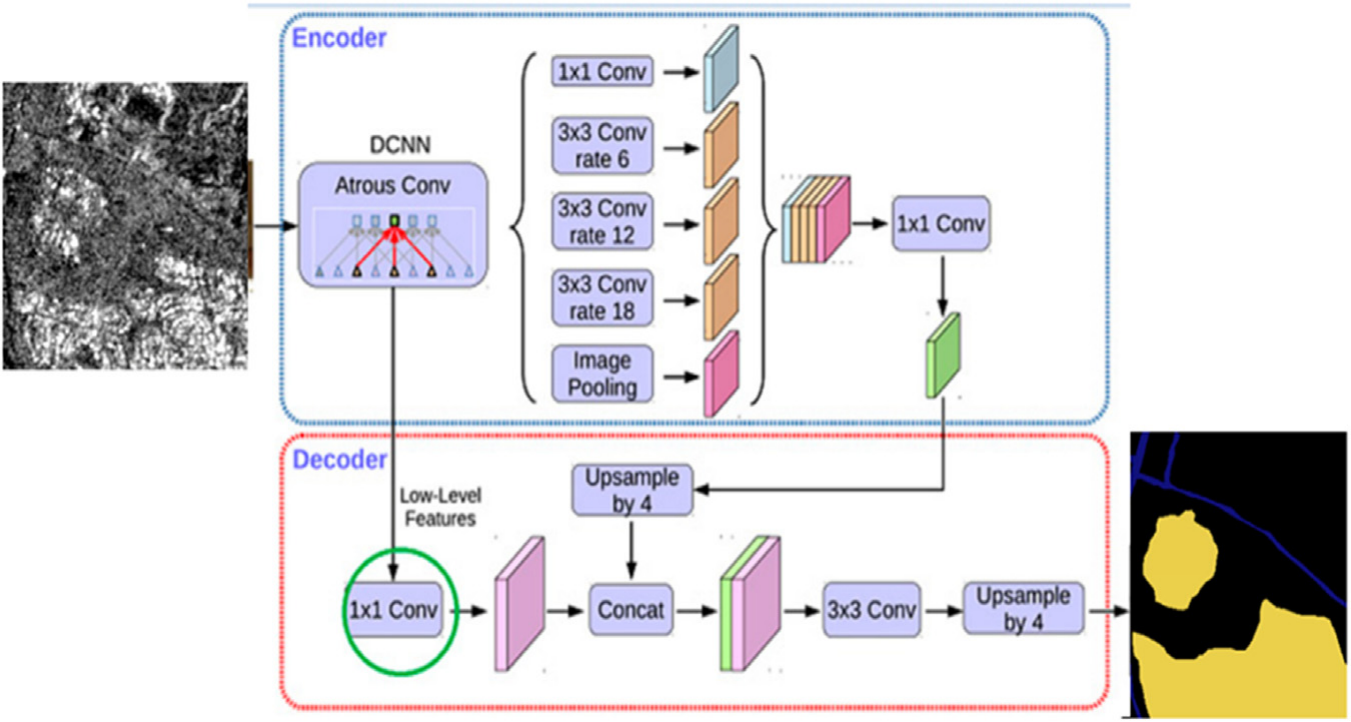
The Deeplabv3+ network [13].

DeepLabv3+ is the most updated version of DeepLab, presented by the same authors in 2018 [13] . In this version, the DeepLabv3 model is included with a simple yet effective decoder module to refine the segmentation results, especially along object boundaries. “DeepLabv3+” is built on the “encoder-decoder” structure, where the “encoder” is accountable for removing low- and high-level semantic data, and the “decoder” further cartels “low-level” and “high-level” features to advance the correctness of segmentation borders and categorize semantic data of diverse pixels. In the encoder, the “DeepLabv3+” model habits “Xception” as the support network and abstracts low and deep features from “Xception”, with the profound features input into the “ASPP module”. The “ASPP module” has four “convolutional layers” with 1, 6, 12, and 18 dilation factors and a global average pooling operation . Furthermore, in this encoder-decoder structure, one can arbitrarily control the resolution of extracted encoder features by atrous convolution to trade off precision and runtime. To further refine the segmentation predictions, the architecture is changed to an encoder-decoder network,

An Improved DeepLabv3+ Network architecture (IDLN) [14] is used in human eye image detection and Segmentation. To segment the target more exceptionally and solve the problem of rough “segmentation boundary”. The “Deeplab v3+” model Ice-Deeplab is the primary network architecture for “sea-ice detection” [15] and automatic pixel-wise “sea-ice Semantic Segmentation”. Ice-Deeplab incorporates a Convolutional Block Attention Module into the unique “Deeplab v3+” architecture to overtly arrest characteristic feature maps. In addition, additional low layer features are added to the novel “decoding branch” to refine the segmentation result. The use of deep learning in “digital image analysis technology” is to advance the “precision and automation of bubble recognition”. The “DeepLab V3+” model integrates multi-scale data, which is well-compatible with “uncertain bubble boundary” data while enhancing the segmentation effect. The lane segmentation detection technique, called Lane-DeepLab [16], built on semantic Segmentation, is used for detecting multi-class lane lines in “unmanned driving scenarios”. Lane-DeepLab uses the “DeepLabv3+” network as the starting point and the “encoder-decoder” structure to produce additional accurate lane line detection results. More explicitly, the atrous convolution is restructured multi-scale by an “attention mechanism”. The DeepLab v3+ model has a good feature extraction ability for processing spatial information such as rural residential houses and ancillary land. By introducing an attention mechanism in the encoding region, the feature extraction accuracy of the model is improved .An “end-to-end space-aware” DeepLab deep learning network named SADNet incorporates a “Space-ware Attentive Residual Module (SARM)” to excerpt rich point cloud features with the assistance of distinguishing spatial relationships amongst points. The automatic gastric cancer segmentation model based on Deeplab v3 + neural network helps achieve better results, improve segmentation accuracy, and save computing resources [17] . DeepLabv3+ is a prevalent semantic segmentation model used across various applications in image segmentation, such as medical imaging, autonomous driving, etc. The Deeplabv3+ network is shown in Fig. 8. ASPP is an essential part of DeepLabV3+ that extracts target feature information using the convolution of multiple receptive fields [18]

The Deeplabv3+ network is used for skin disease diagnosis with three stages: segmentation, feature extraction, and classification [19]. The network can be used for cracks on tunnel linings [20]. and visual scene understanding of soil types [21]. The Deeplab V3+ semantic segmentation model developed using the framework of ResNet-10 can be used for the study of the respiratory rate (RR) of dairy cows [22]. DeepLab v3+ models with a ResNet-50 backbone and a fuzzy connectedness analysis module for epidermal layer segmentation. Deeplabv3+ is used to semantically segment Synthetic aperture radar (SAR), which provides rich information about the Earth’s surface under all weather and day-and-night conditions and is applied in many relevant fields [23]. A “feature cross attention module (FCA)” [24] is used in the Deeplabv3+ model. The cross-attention network comprises two branches: the low branch is utilized to excerpt “low-level spatial data”, and the deep branch is used to excerpt “high-level context features”.

## Method details

### Methodology

**Fig. 9:**
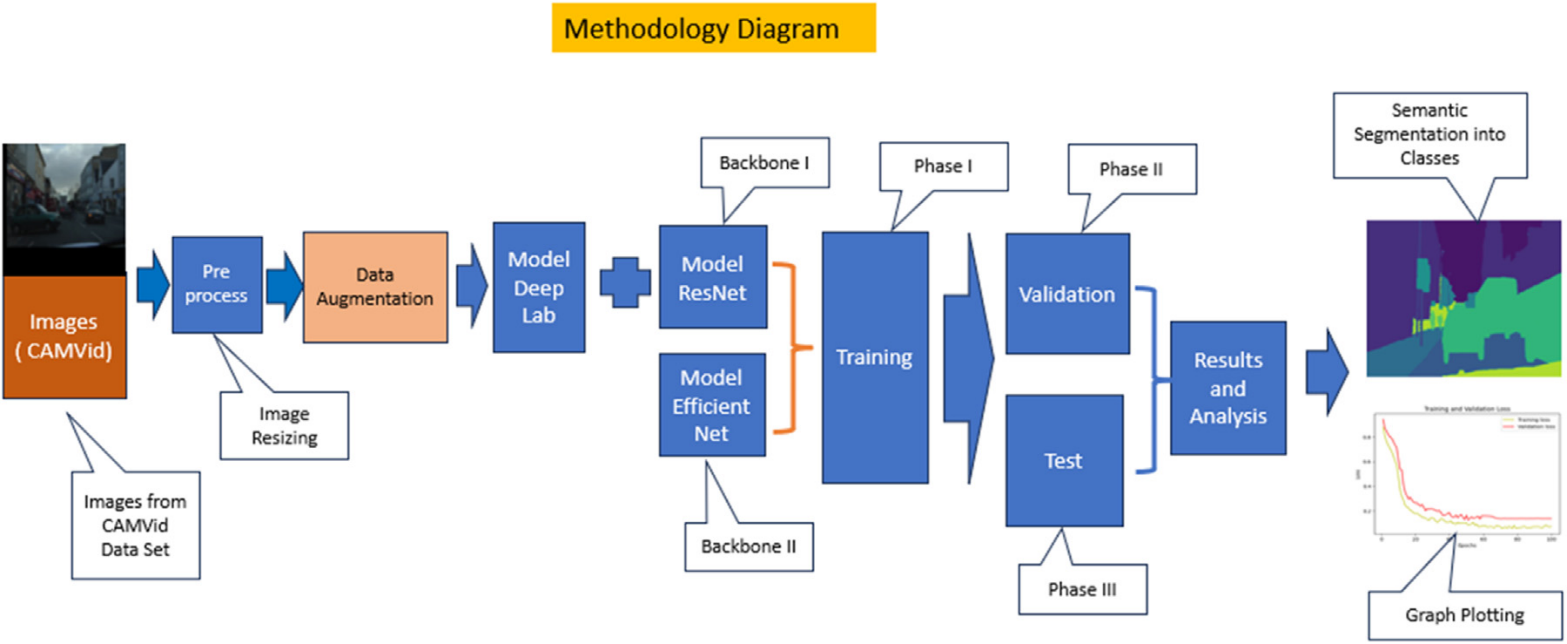
Methodology diagram.

The Methodology Diagram in Fig. 9 shows the hybrid architecture’s implementation method. The blocks and the flow in the Methodology Diagram are explained below.

i. Images: The images from the CAMVid data set are used for evaluation.
ii. Pre-process: Pre-processing of the images includes processing images in the same way as the backbone, as defined by the library. This step also includes the resizing of images, learning rate =0.00001 and clipnorm=1.0
iii. Data Augmentation: The images are augmented for horizontal flip, shift, scale, rotation, crop, etc.
iv. Model DeepLab V3+: The Model DeepLab V3+ model is used for semantic Segmentation.
v. Backbones: ResNet and EfficientNetv2 are used as the backbone.
vi. Training: The combined models are trained for 100 Epochs for the training set of images
vii. Validation and Testing: The hybrid models are validated and tested for 100 Epochs for the validation and testing data set of images, respectively.

#### Data set

**Fig. 10:**
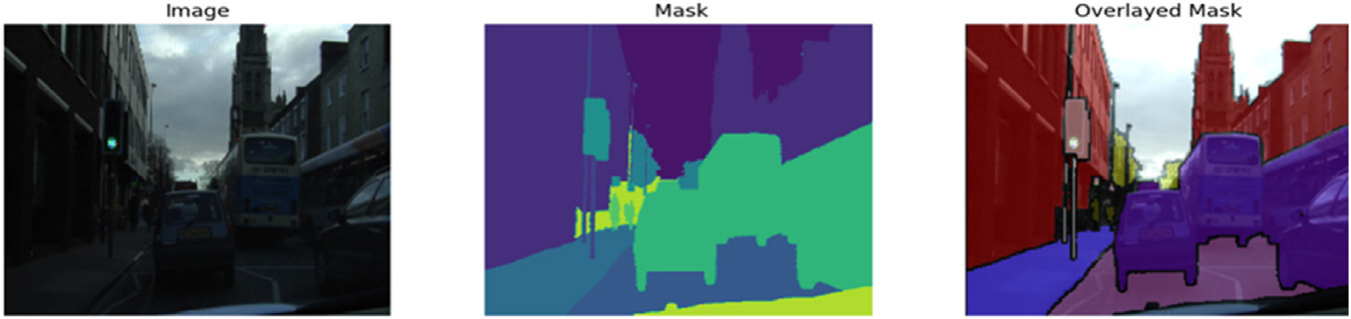
Sample image, mask and overlayed mask.

The CamVid (Cambridge-driving Labelled Video Database) [25] is used for training, validating and testing the different models. The data set provides ground truth labels associating each pixel with one of 11 semantic classes. This dataset is often used in (real-time) semantic segmentation research. The dataset comprises 367 training pairs (image and mask), 101 validation, and 233 test pairs. This data set has eleven classes like ‘sky’, ‘building’, ‘pole’, ‘road’, ‘pavement’, ‘tree’, ‘sign-symbol’, ‘fence’, ‘car’, ‘pedestrian’, ‘bicyclist’ and ‘unlabeled’. The twelfth class contains unlabeled data, which are ignored while training. All images have 360 pixels in height and 480 pixels in width. Fig. 10 Shows the dataset’s sample image, mask and overlayed mask.

The colourmap for the 11 classes for the CamVid data set is shown in Table 1 From Sky to the Bicyclist.

**Table 1:** Colormap CamVid data set.

Id	RGB values	Class
0	(128, 128, 128)	Sky
1	(128,0,0)	Building
2	(192, 192, 192)	Pole
3	(128, 64, 128)	Road
4	(60, 40, 222)	Pavement
5	(128, 128, 0)	Tree
6	(192,128, 28)	Sign Symbol
7	(64, 64, 128)	Fence
8	(64, 0, 128)	Car
9	(64, 64, 0)	Pedestrian
10	(0, 128,192)	Bicyclist

#### Semantic segmentation using deeplab V3+

KerasCV has integrated DeepLabv3+ into its library [26]. KerasCV is a Keras model implementing the DeepLabV3+ architecture for semantic Segmentation. It allows seamless customization of models and other training pipelines across major computer vision domains, such as “classification”, “object detection” and “semantic Segmentation”. ImageNet pre-trained backbones ResNet50, ResNet50V2 and EfficientNetv2 are feature extractors for fine-tuning the DeepLabv3+ Model.

##### DeepLab V3+ backbones resnet50v2

**Fig. 11:**
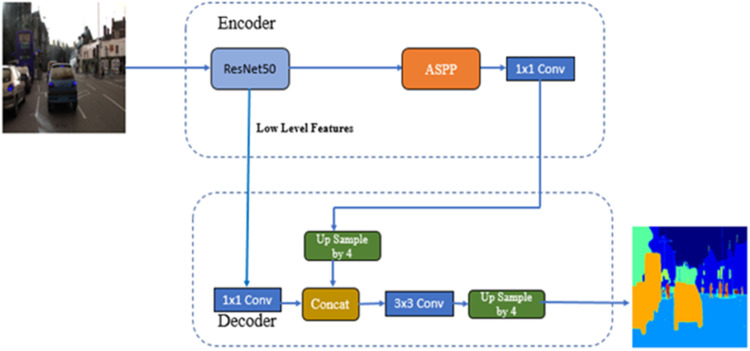
Block diagram of DeepLab V3+ with backbones ResNet50V2.

The hyper-parameter Configurations for the KerasCV DeepLabv3+ model with ResNet50V2 [27] as the backbone include an image size of (512, 512, batch size of 2, brightness factor of 0.2 and contrast factor of 0.2. Since the CamVid data set is used, the number of classes is 11. The training configuration class handles the training hyper-parameters, such as model backbone, the number of epochs, learning rate, etc. Fig. 11 shows the Block Diagram of DeepLab V3+ with backbones ResNet50V2. In this architecture, the ResNet50V2 model is part of the encoder. The model parameters for this architecture are summarized in Table 2 Which include the Total parameters, Trainable parameters and non-trainable parameters.

**Table 2:** DeepLab V3+ backbones ResNet50V2 model parameters.

Total params:	17,833,036
Trainable params:	17,798,252
Non-trainable params:	34,784

##### DeepLab V3+ backbones resnet50v2 with squeeze excitation module

**Fig. 12:**
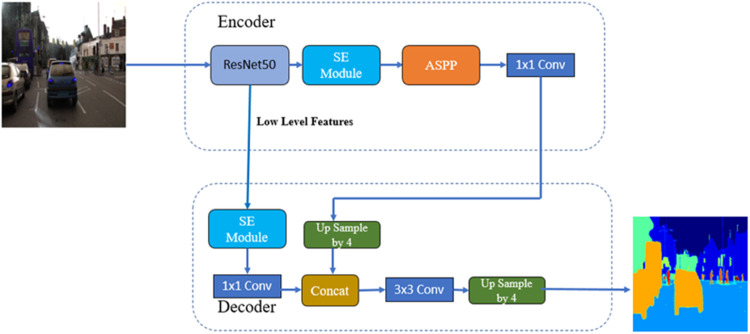
Block diagram of DeepLab V3+ with backbones ResNet50V2 and SE module.

The “Squeeze-and-Excitation (SE)” module calibrates “channel-wise feature” retorts by overtly modelling channel “interdependences” [27]. SE module gives significant enhancements in performance for prevailing Convolutional Neural Networks at a trivial additional computational cost. The features are first conceded by a “squeeze operation”, which generates a “channel descriptor” by accumulating feature maps across their “spatial dimensions”. The purpose of this “descriptor” is to create an entrenching of the universal distribution of “channel-wise feature responses”, permitting data from the comprehensive receptive field of the network to be used by all its layers. The “aggregation” is trailed by an “excitation operation”, which takes the form of a simple “self-gating mechanism” that takes the entrenching as input and produces a group of “per-channel modulation weights”. These weights are used for the feature maps to make the output of the “SE block”, which can be suckled directly into succeeding layers of the network. Constructing an “SE network” by piling a collection of “SE blocks” is possible. Moreover, these “SE blocks” can also be used as a drop-in additional for the unique chunk at a “range of depths” in the network. The structure of the “SE block” is simple and can be used directly in existing state-of-the-art architectures by replacing components with their SE counterparts. SE blocks are also computationally lightweight and impose only a slight increase in model complexity and computational burden. Fig. 12 shows the Block Diagram of DeepLab V3+ with backbones ResNet50V2 and SE Module. In this architecture, the ResNet50V2 model and SE Module are part of the encoder. The model parameters for this architecture are summarized in Table 3 includes the total parameters, trainable parameters, and non-trainable parameters.

**Table 3:** DeepLab V3+: backbones EfficientNetv2 with squeeze excitation module model parameters.

Total params:	17,177,484
Trainable params:	17,143,052
Non-trainable params:	34,432

##### DeepLab V3+ backbones resnet50v2 with CBAM module

**Fig. 13:**
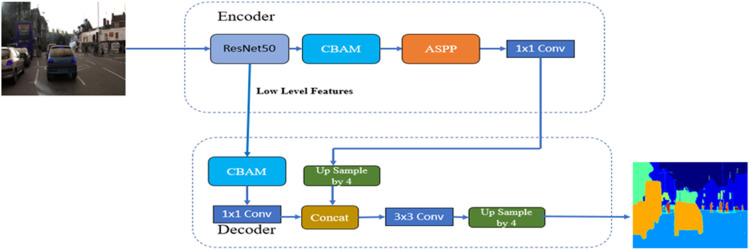
Block Diagram of DeepLab V3+ with backbones ResNet50V2 and CBAM Module.

The “Convolutional Block Attention Module (CBAM)” [28] has two successive sub-modules: channel and spatial. The in-between feature map is adaptively improved through the CBAM module at each “convolutional block” of deep networks. Fig. 13 shows the Block Diagram of DeepLab V3+ with backbones ResNet50V2 and CBAM Module. In this architecture, the ResNet50V2 model and CBAM Module are part of the encoder. The model parameters for this architecture are summarized in Table 4 DeepLab V3+: backbones EfficientNetv2 with CBAM Module model parameters, including the Total, Trainable, and Non-trainable parameters.

**Table 4:** DeepLab V3+: backbones EfficientNetv2 with CBAM Module model parameters.

Total params:	17,518,862
Trainable params:	17,484,430
Non-trainable params:	34,432

##### DeepLab V3+ backbones efficientnetv2

**Fig. 14:**
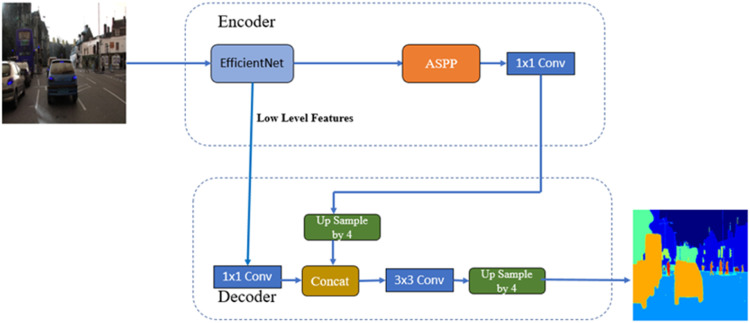
Block Diagram of DeepLab V3+ with backbones efficientNetv2.

Fig. 14 shows the Block Diagram of DeepLab V3+ with EfficientNetv2 as the backbone. In this architecture, the EfficientNetv2 model is part of the encoder. The model parameters for this architecture are summarized in Table 5 DeepLab V3+: backbones EfficientNetv2 model parameters. These include the Total, Trainable, and Non-trainable parameters.

**Table 5:** DeepLab V3+: backbones efficientNetv2 model parameters.

Total params:	4016,363
Trainable params:	3987,636
Non-trainable params:	28,727

##### DeepLab V3+ with backbone of efficientnetv2 and SE module

**Fig. 15:**
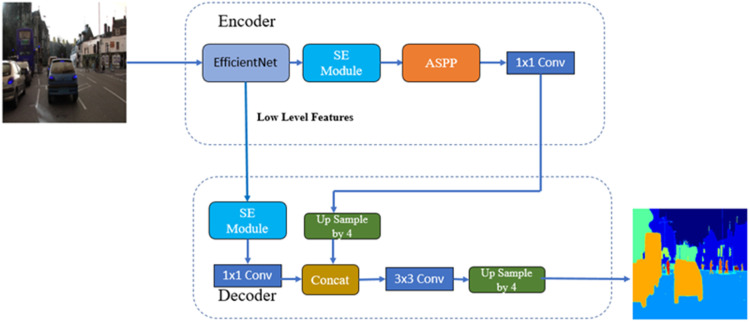
Block diagram of DeepLab V3+ with backbones efficientNetv2 and SE module.

Fig. 15 shows the Block Diagram of DeepLab V3+ with EfficientNetv2 as the backbone and SE Module. In this architecture, the EfficientNetv2 model and SE Module are part of the encoder. The model parameters for this architecture are summarized in Table 6, including the Total, Trainable, and Non-trainable parameters.

**Table 6:** DeepLab V3+: backbones efficientNetv2 model parameters.

Total params:	4026,603
Trainable params:	3997,876
Non-trainable params:	28,727

##### DeepLab V3+ with backbones efficientnetv2 and CBAM module

**Fig. 16:**
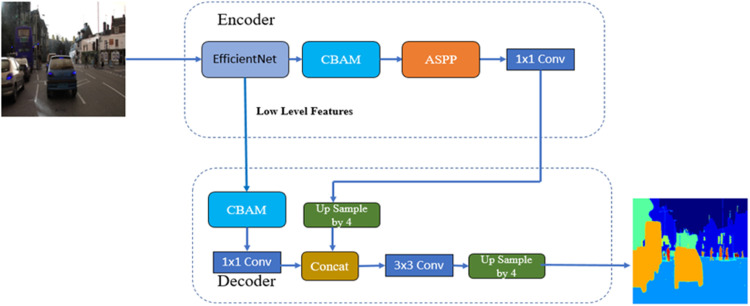
Block diagram of DeepLab V3+ with backbones EfficientNetv2 and CBAM Module.

Fig. 16 shows the Block Diagram of DeepLab V3+ with EfficientNetv2 as the backbone and CBAM Module. In this architecture, the EfficientNetv2 model and CBAM Module are part of the encoder.

The model parameters are summarized in Table 7, which includes the Total parameters, Trainable parameters and the non-trainable parameters.

**Table 7:** DeepLab V3+ backbones EfficientNetv2 model parameters.

Total params:	8043,133
Trainable params:	8012,870
Non-trainable params:	30,263

### Evaluation metrics

For semantic image segmentation, commonly used evaluation metrics include “Mean Pixel Accuracy (MPA)”, “Intersection over Union (IoU)”, Categorical Accuracy and “Mean Intersection Over Union (mIoU)”. These metrics are explained below.

#### Mean pixel accuracy (mPA)

MPA represents the fraction of appropriately “classified pixels” amongst all pixels in a “complete image” [29]. The formulas of mPA are as follows,

$$mPA=\frac{TP}{TP+FP}$$

The model envisages a positive example where TP represents the authentic case, but the authentic example is also positive. FP is for “false positives”, where the model envisages a “positive example”, but the authentic example is “negative”.

#### Intersection over union (IoU)

IoU is a metric often used in segmentation problems to assess the model’s accuracy. It provides a more intuitive basis for accuracy that is not biased by the (unbalanced) percentage of pixels from any particular class. Given two segmentation masks, `A` and `B`, the IoU is defined as follows.

$$IoU=\frac{\left|A\cap B\right|}{\left|A\cup B\right|}$$

When multiple classes and inferences exist, we assess the model’s performance by computing the mean IoU. The function mean_iou below computes the mean IoU that only considers the classes in the ground truth mask or the predicted segmentation map (sometimes called class-wise mean IoU). This computation better represents the metric since it only considers the relevant classes.

#### Categorical accuracy

Categorical accuracy [30] calculates how often predictions match one-hot labels. It measures the proportion of correctly classified pixels in an image. It gives a simple percentage indicating how accurately the model predicts the categories of pixels in the entire image. Accuracy considers all class errors, but categorical accuracy only finds the extent of errors in a particular class. This metric generates two local variables, total and count, that calculate the frequency with which the predicted value matches the actual value. This frequency is eventually returned as categorical accuracy: an idempotent operation that divides the total by count.

#### Mean intersection over union (mIoU)

In essence, MeanIoU emphasizes the accuracy of identifying specific object boundaries, while Categorical Accuracy gives a broad overview of overall pixel-level correctness. MeanIoU is defined as

$$mIoU=\frac{TP}{FP+FN+TP}$$

The model envisages a positive example where TP represents the authentic example, but the authentic example is also positive. FP is for “false positives”, where the model envisages a “positive example”, but the authentic example is “negative”. FN stands for “false negative” case, a positive case when the model envisages a negative case.

## Method validation

### Results and discussion

The results for the DeepLab V3+ with the backbone ResNet50 Model, DeepLab V3+: backbone ResNet50 with Squeeze and excitation module Model, and DeepLab V3+: backbone EfficientNet and DeepLab V3+: backbone EfficientNet with Squeeze and excitation module Model are discussed below. The “training and validation” phase metrics are discussed, such as loss, accuracy, and mean IoU for the above models.

#### DeepLab V3+: backbone resnet50

**Fig. 17:**
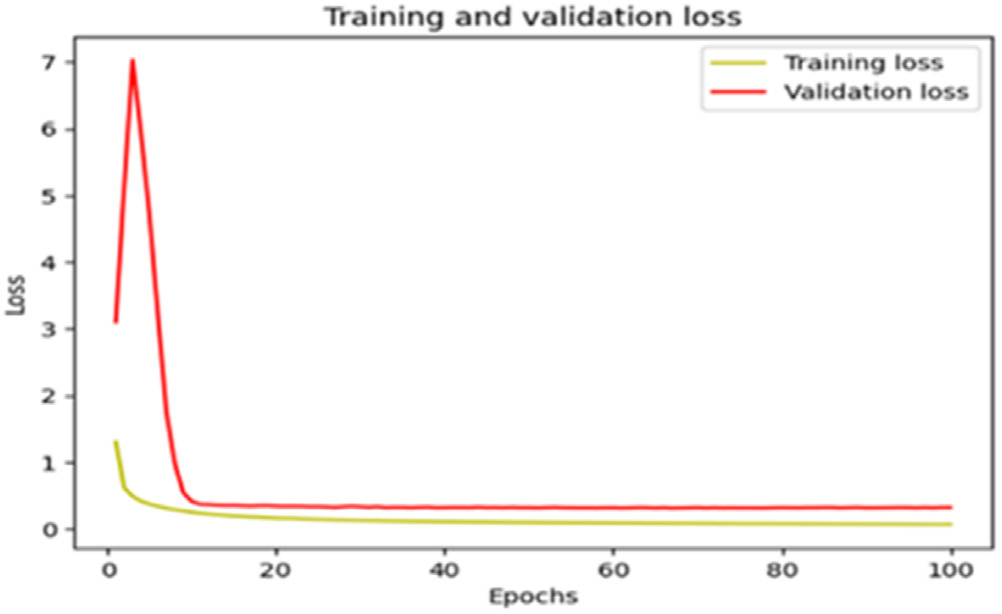
“Training and validation loss”.

**Fig. 18:**
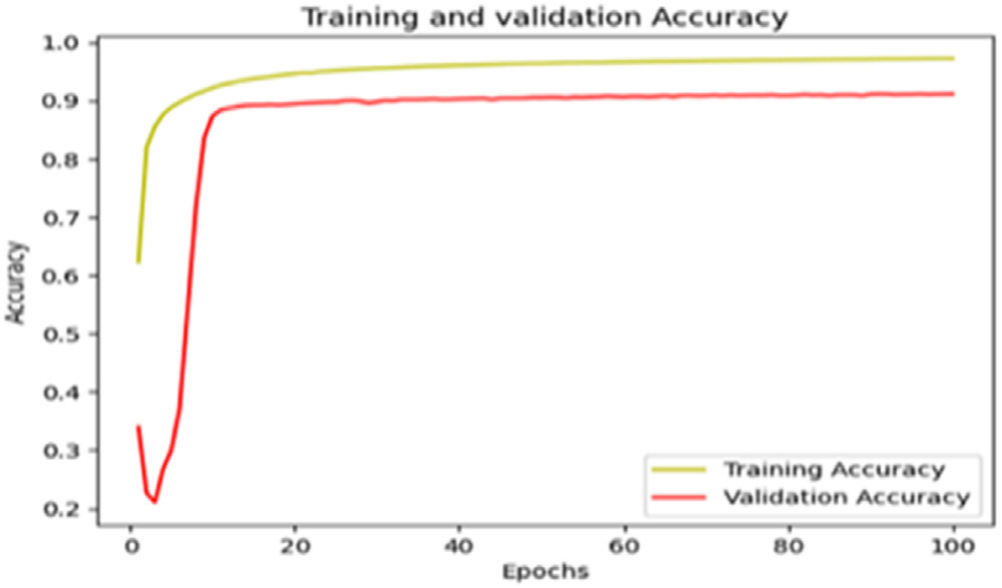
“Training and validation accuracy”.

The “Training and validation” loss for the “DeepLab V3+ model” with the backbone of ResNet50 architecture is shown in Fig. 17. The “Training and validation Accuracy” for the DeepLab V3+ model with the backbone of ResNet50 architecture is indicated in Fig. 18.

**Fig. 19:**
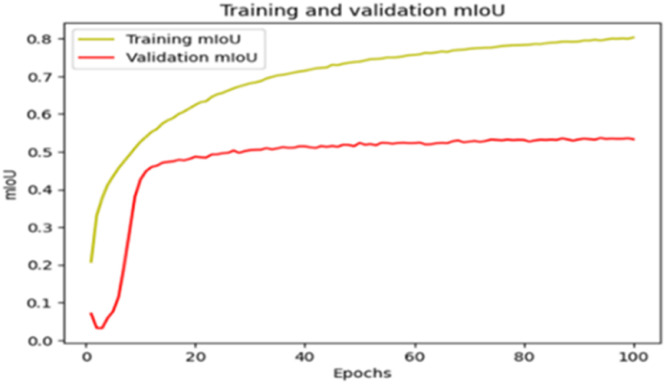
“Training and validation” mIoU.

**Fig. 20:**
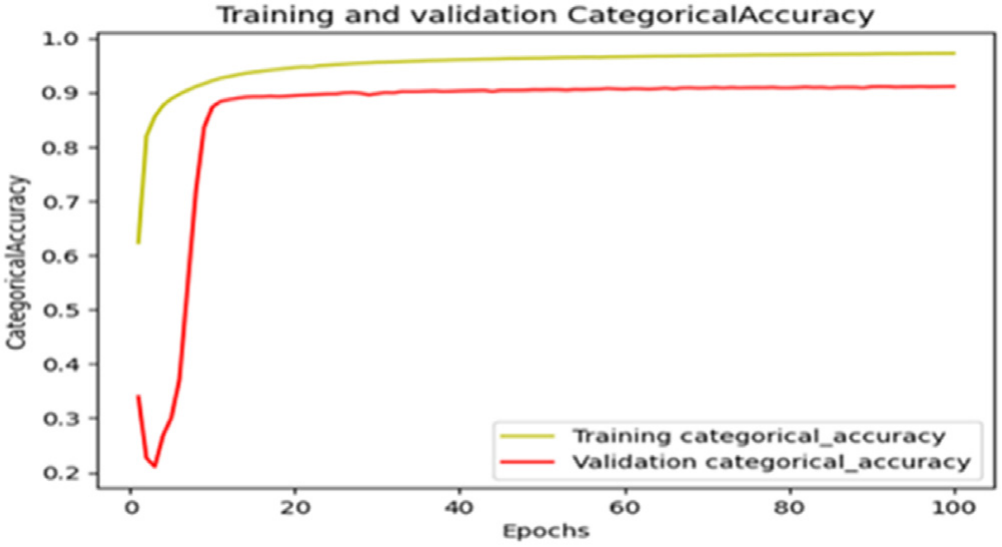
Training and validation categorical accuracy.

The “Training and validation” mIoU for the “DeepLab V3+” model with the backbone of ResNet50 architecture is shown in Fig. 19. The “Training and validation” Categorical Accuracy are indicated in Fig. 20.

**Fig. 21:**
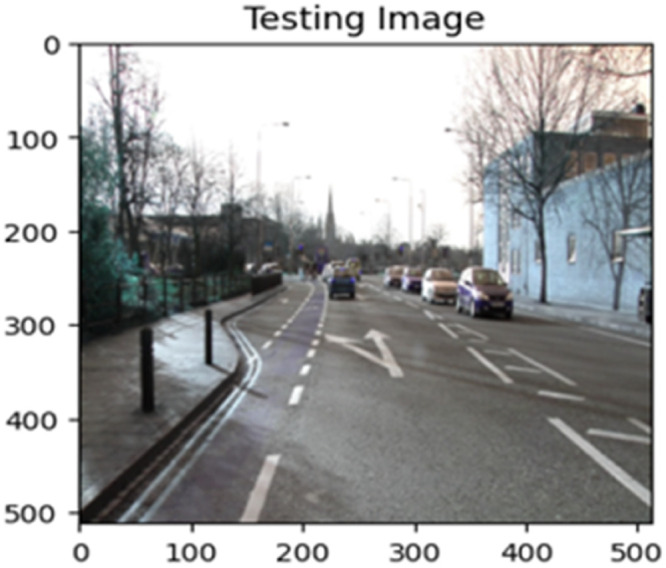
Testing image.

**Fig. 22:**
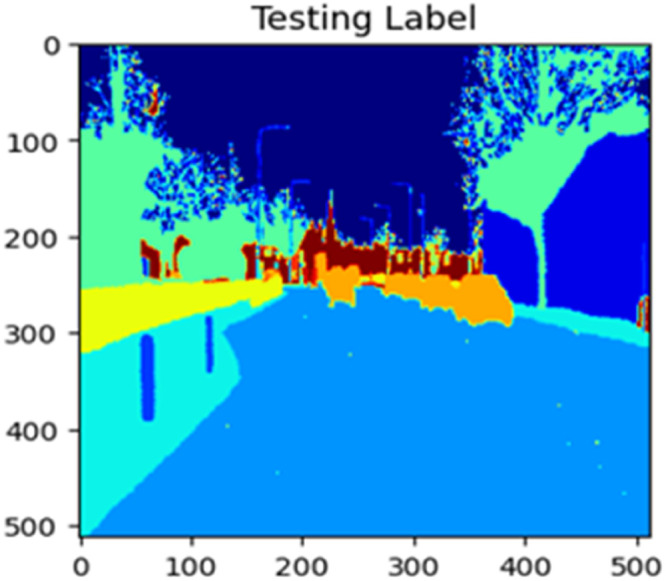
Testing label.

**Fig. 23:**
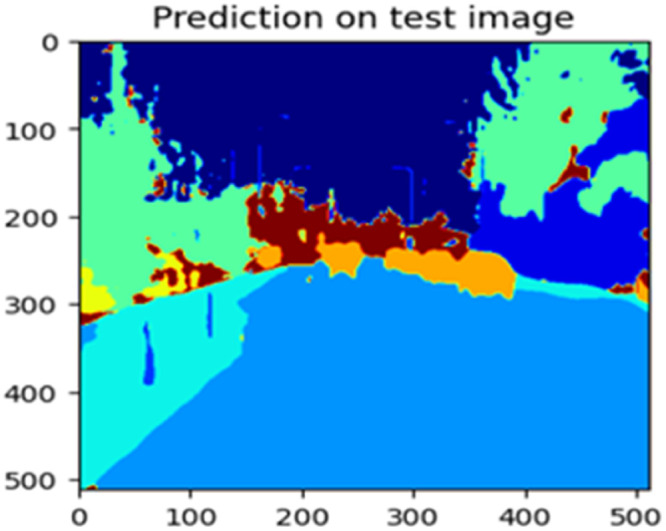
Prediction on test image.

The “testing image”, “testing label”, and prediction of the “test image” for the DeepLab V3+ model with the backbone of ResNet50 architecture are indicated in Fig. 21, Fig. 22, Fig. 23 correspondingly.

The summary of the metrics for the DeepLab V3+ model with the backbone of ResNet50 architecture is shown in Table 8.

**Table 8:** Summary of metrics after 100 Epochs.

Phase	Accuracy	Loss	Categorical Accuracy	Mean IoU
Training	0.9725	0.0699	0.9725	0.8056
Validation	0.9109	0.3247	0.9109	0.5330

#### DeepLab V3+: backbone resnet50 with squeeze excitation module

**Fig. 24:**
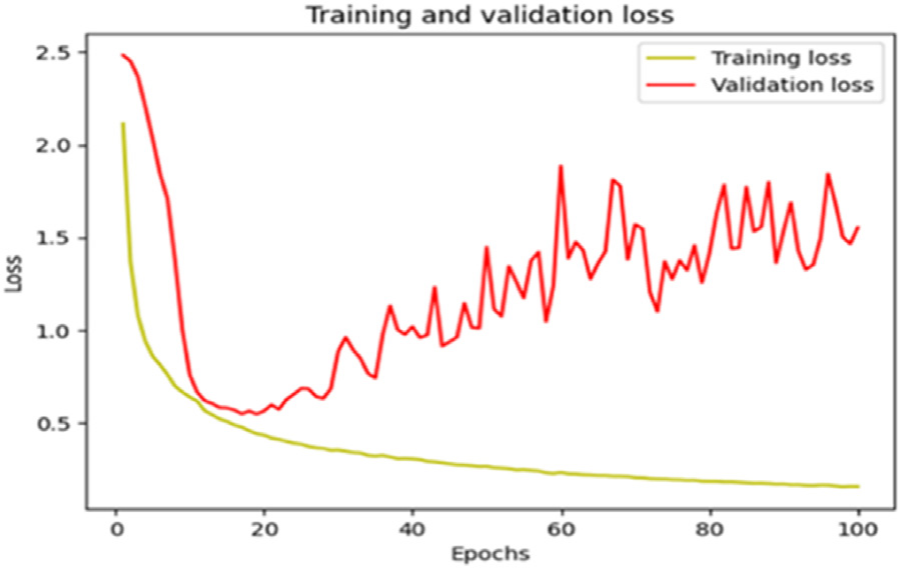
“Training and validation loss”.

**Fig. 25:**
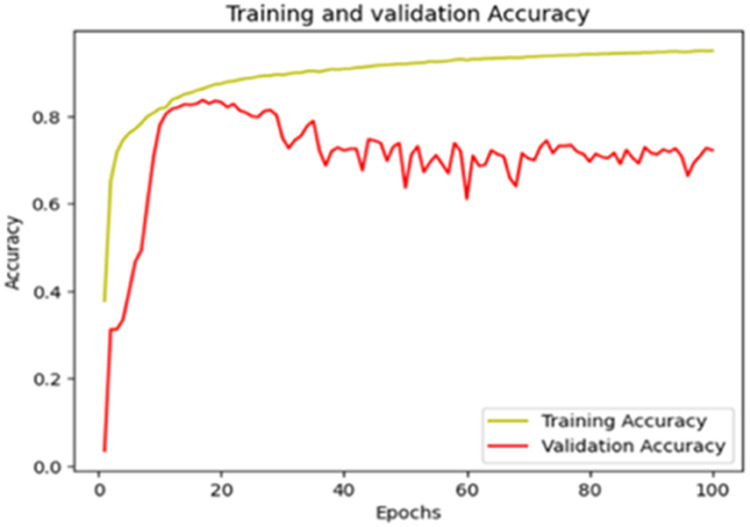
“Training and validation accuracy”.

The “Training and validation loss” for the “DeepLab V3+” model with the backbone of EfficientNetv2 architecture is indicated in Fig. 24. The “Training and validation Accuracy” for the “DeepLab V3+” model with the backbone of EfficientNetv2 architecture is indicated in Fig. 25.

**Fig. 26:**
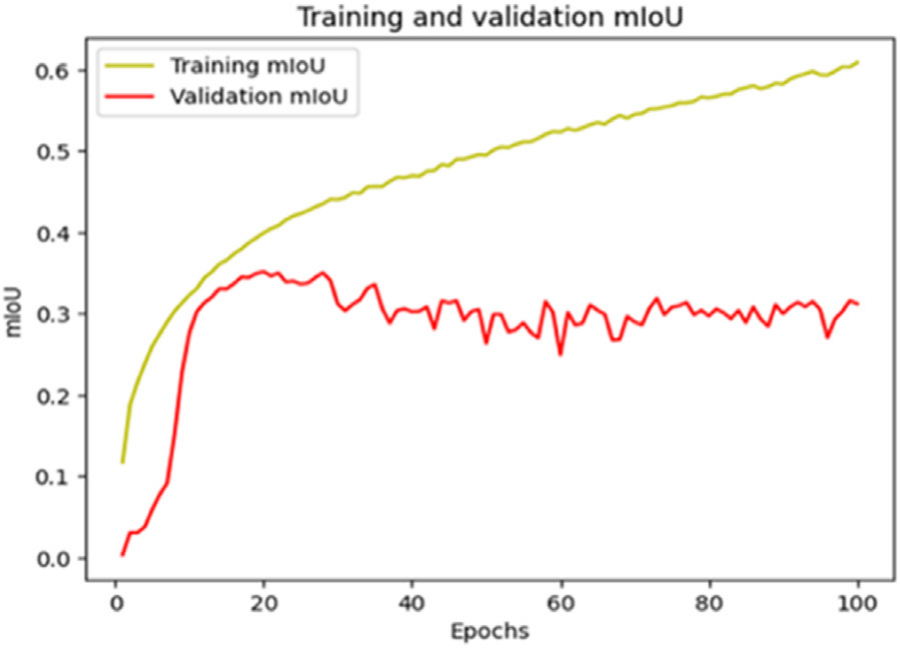
“Training and validation” mIoU.

**Fig. 27:**
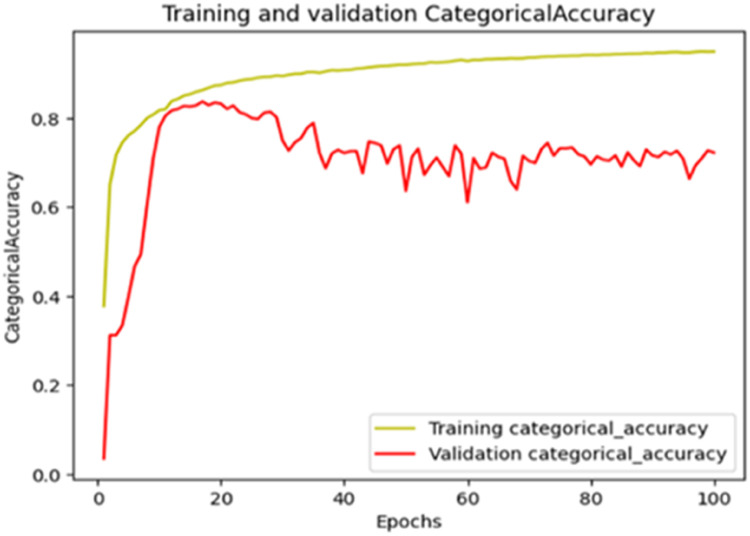
“Training and validation Categorical Accuracy”.

The Training and validation mIoU for the DeepLab V3+ model with the backbone of EfficientNetv2 architecture is indicated in Fig. 26. The “Training and validation Categorical Accuracy” are indicated in Fig. 27.

**Fig. 28:**
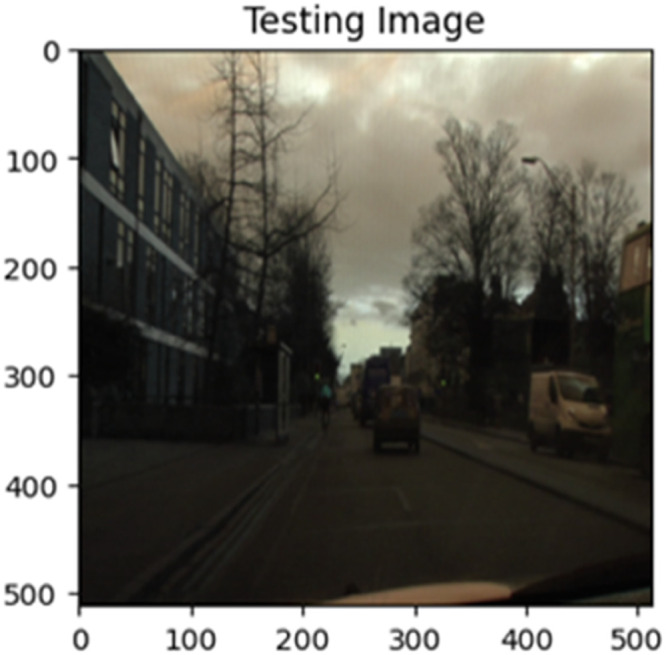
Testing image.

**Fig. 29:**
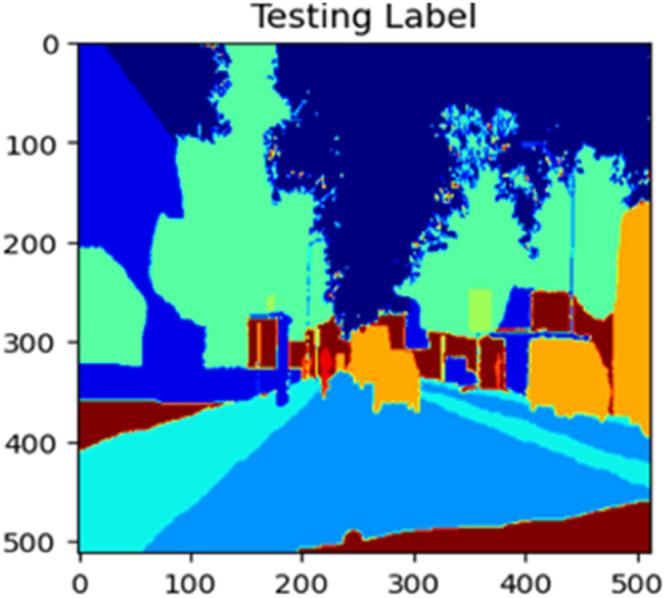
Testing label.

**Fig. 30:**
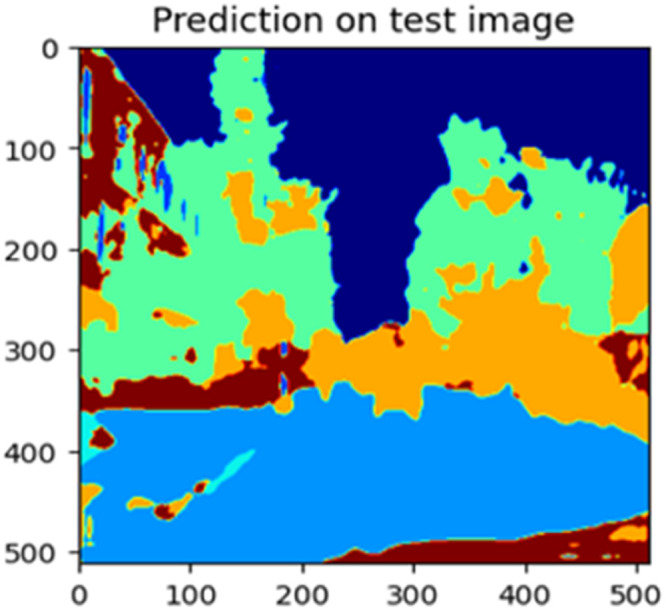
Prediction on test image.

The “testing image”, “testing label”, and prediction of the “test image” for the DeepLab V3+ model with the backbone of EfficientNet with Squeeze Excitation module architecture is shown in Fig. 28, Fig. 29, Fig. 30 respectively.

The summary for the DeepLab V3+ model with the backbone of EfficientNetv2 architecture is shown in Table 9.

**Table 9:** Summary of metrics after 100 Epochs.

Phase	Accuracy	Loss	Categorical Accuracy	Mean IoU
Training	0.9501	0.1556	0.9501	0.6124
Validation	0.7222	1.5531	0.7222	0.3121

#### DeepLab V3+ backbones resnet50v2 with CBAM module

**Fig. 31:**
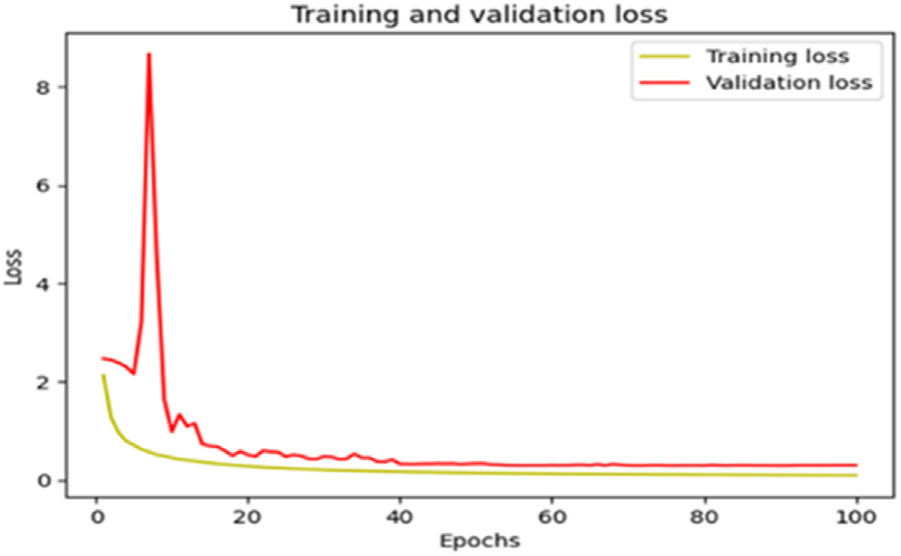
Training and validation loss.

**Fig. 32:**
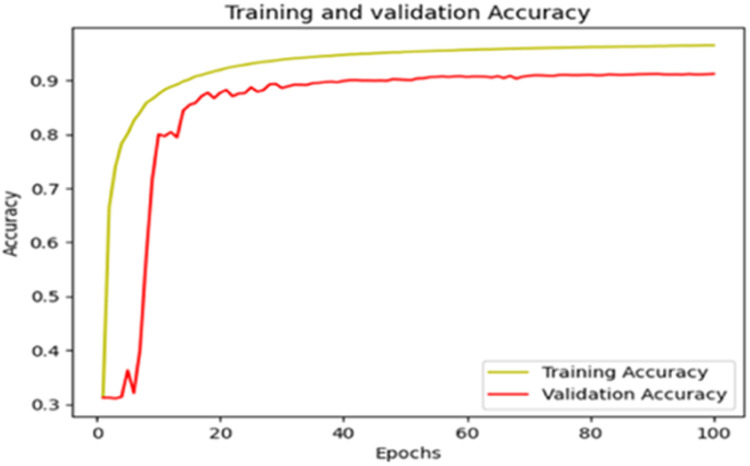
Training and validation” accuracy.

The “Training and validation loss” for the “DeepLab V3+” model with the backbone of EfficientNetv2 architecture is indicated in Fig. 31. The “Training and validation Accuracy” for the “DeepLab V3+” model with the backbone of EfficientNetv2 architecture is indicated in Fig. 32

**Fig. 33:**
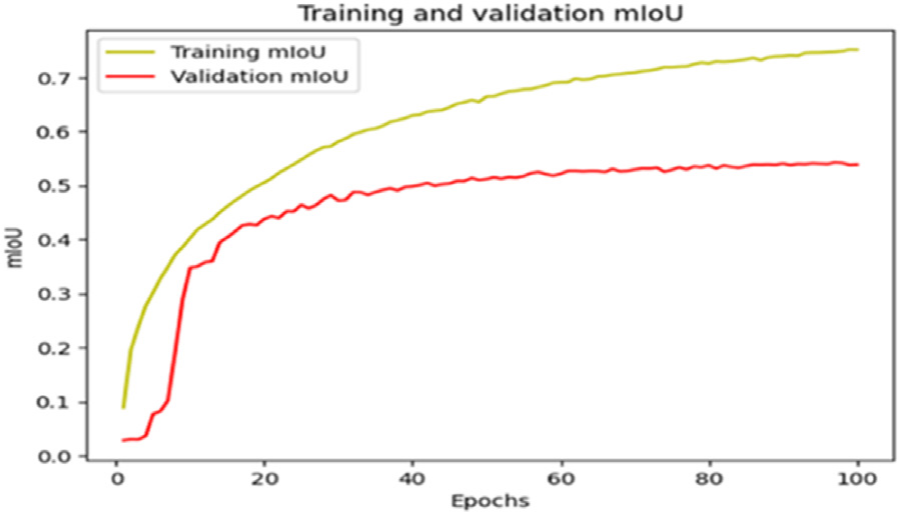
“Training and validation mIoU”.

**Fig. 34:**
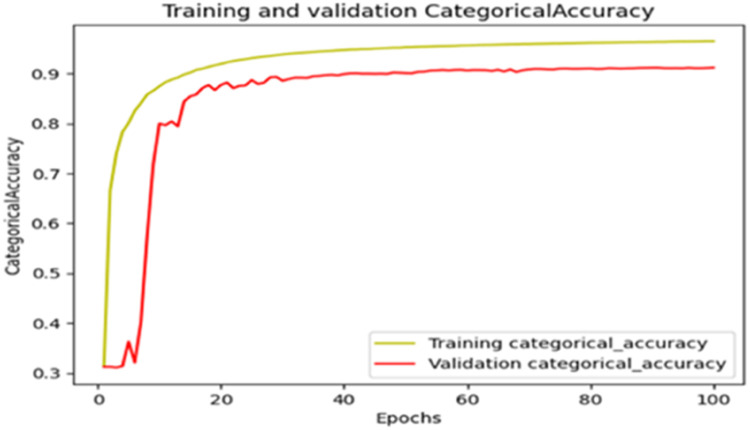
“Training and validation categorical accuracy”.

The “Training and validation” mIoU for the DeepLab V3+ model with the backbone of EfficientNetv2 architecture is indicated in Fig. 33. The “Training and validation Categorical Accuracy” are indicated in Fig. 34.

**Fig. 35:**
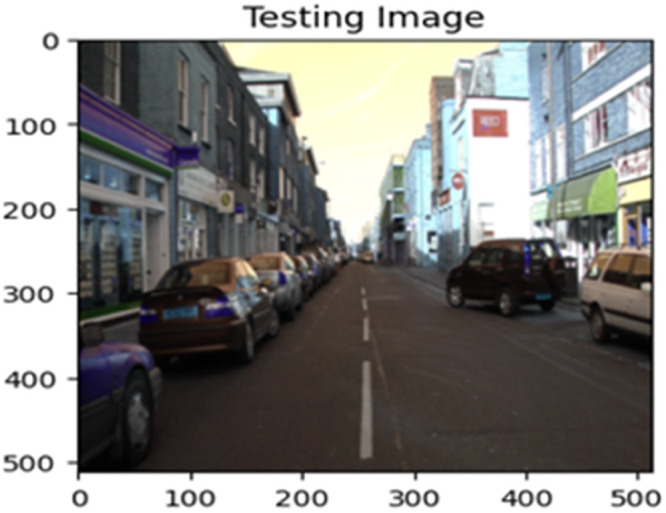
Testing Image.

**Fig. 36:**
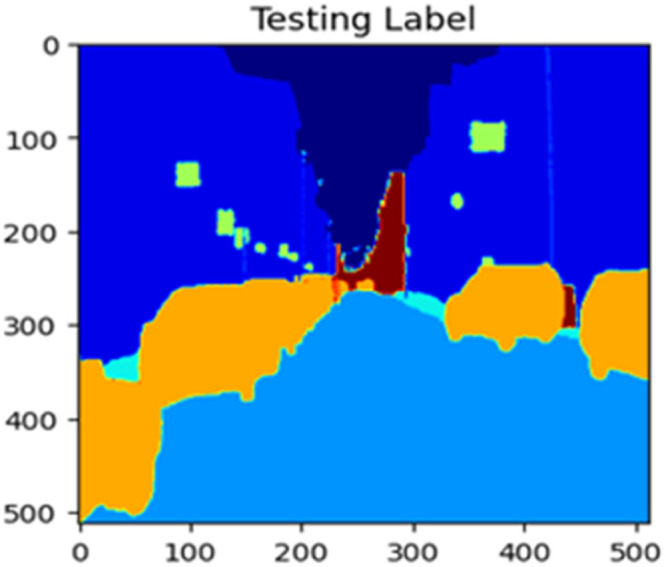
Testing Label.

**Fig. 37:**
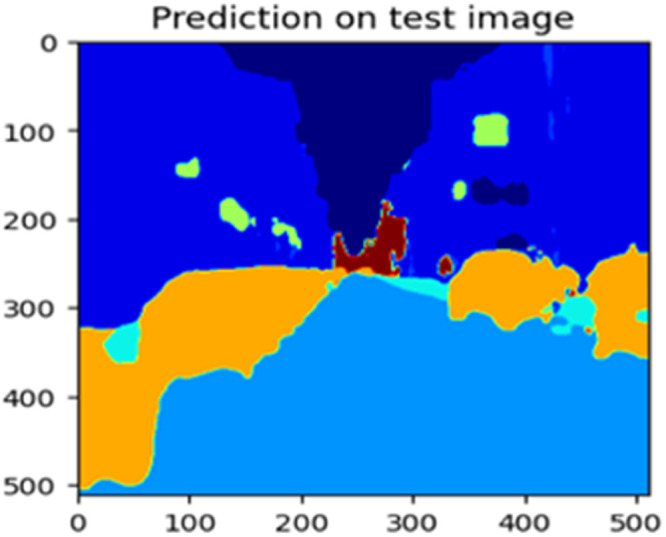
Prediction on test image.

The “testing image”, “testing label”, and prediction of the “test image” for the DeepLab V3+ model with the backbone of EfficientNet with Squeeze Excitation module architecture is indicated in Fig. 35, Fig. 36, Fig. 37 correspondingly.

The summary for the DeepLab V3+ model with the backbone of EfficientNetv2 architecture is shown in Table 10.

**Table 10:** Summary of metrics after 100 Epochs.

Phase	Accuracy	Loss	Categorical Accuracy	Mean IoU
Training	0.9664	0.0905	0.9664	0.7554
Validation	0.9119	0.3005	0.9119	0.5387

#### DeepLab V3+ backbones efficientnetv2

**Fig. 38:**
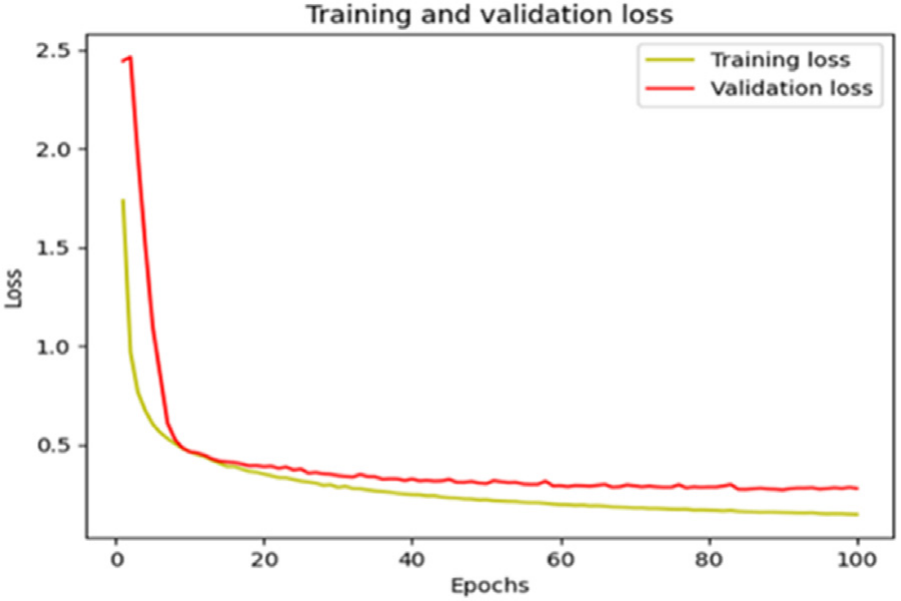
Training and validation” loss.

**Fig. 39:**
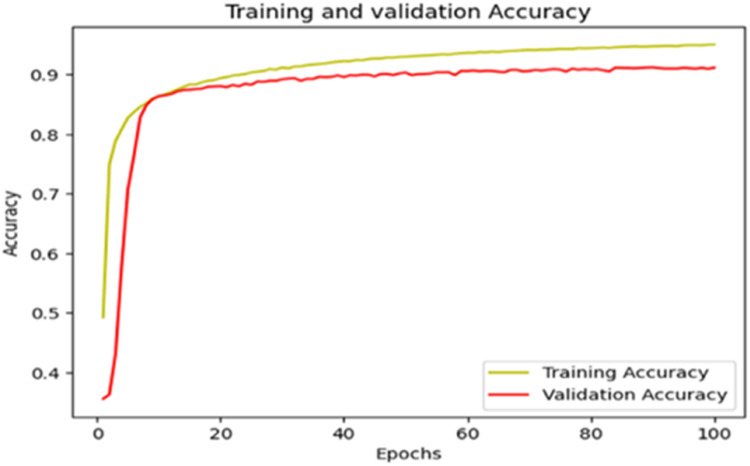
Training and validation” accuracy.

The “Training and validation” loss for the “DeepLab V3+ model” with the backbone of EfficientNetv2 architecture is indicated in Fig. 38. The “Training and validation” Accuracy for the DeepLab V3+ model with the backbone of EfficientNetv2 architecture is indicated in Fig. 39.

**Fig. 40:**
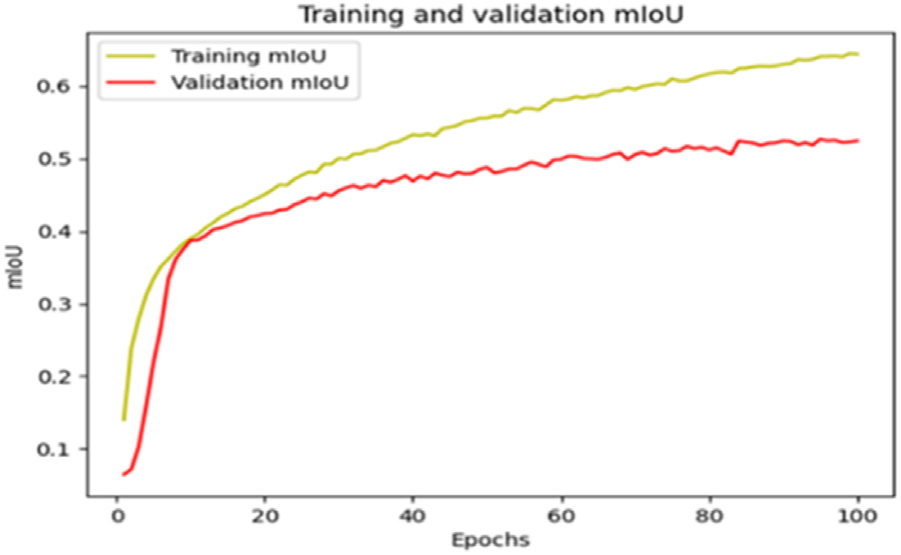
Training and validation” mIoU.

**Fig. 41:**
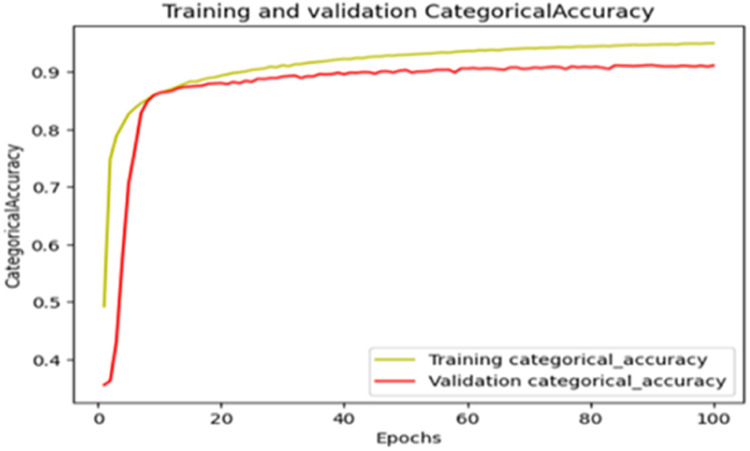
Training and validation” categorical accuracy.

The “Training and validation” mIoU for the DeepLab V3+ model with the backbone of EfficientNetv2 architecture is indicated in Fig. 40. The “Training and validation” Categorical Accuracy is indicated in Fig. 41.

**Fig. 42:**
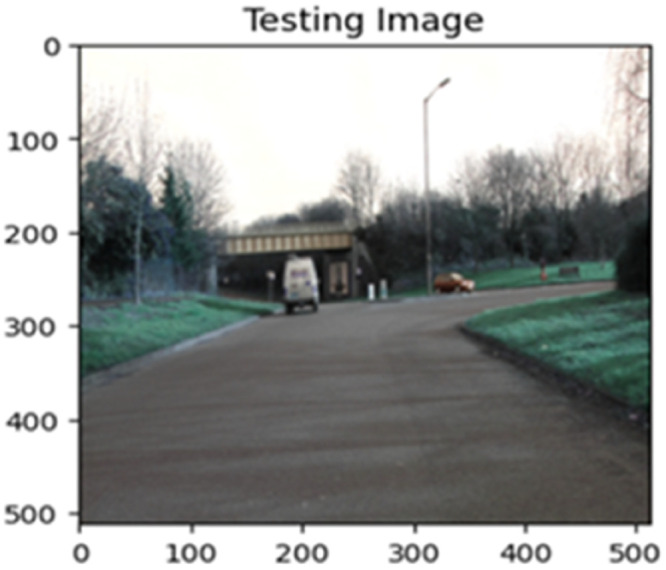
Testing image.

**Fig. 43:**
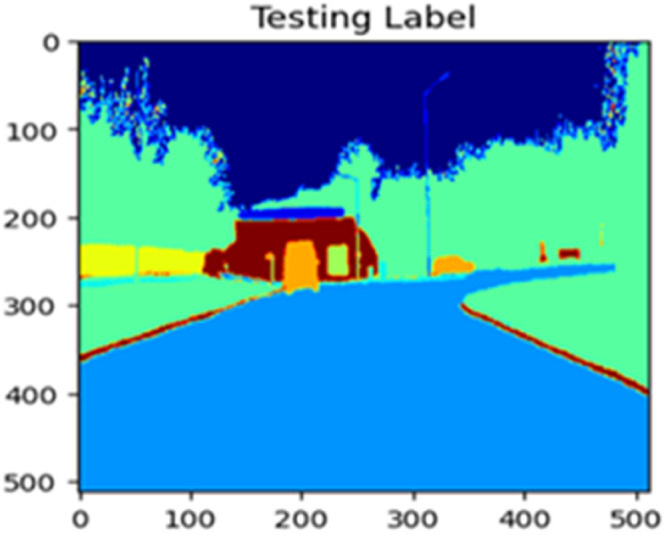
Testing label.

**Fig. 44:**
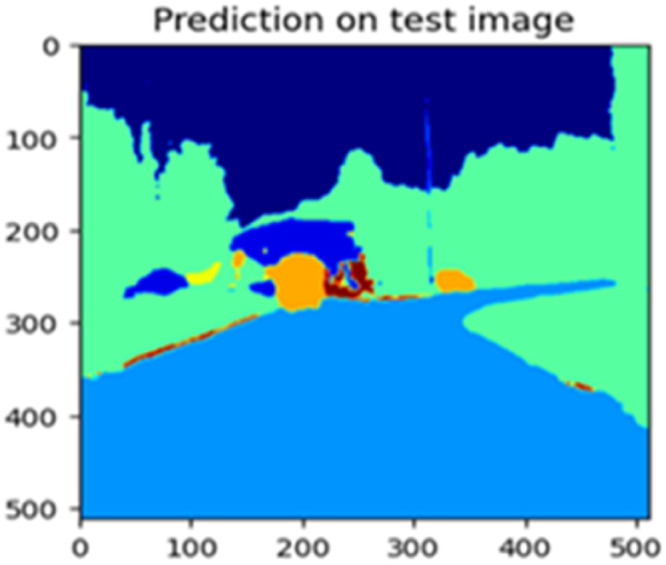
Prediction on test image.

The “testing image”, “testing label”, and prediction of the “test image” for the DeepLab V3+ model with the backbone of EfficientNet with Squeeze Excitation module architecture is indicated in Fig. 42, Fig. 43, Fig. 44 correspondingly.

The summary for the DeepLab V3+ model with the backbone of EfficientNetv2 architecture is shown in Table 11.

**Table 11:** Summary of metrics after 100 Epochs.

Phase	Accuracy	Loss	Categorical Accuracy	Mean IoU
Training	0.9491	0.1487	0.9491	0.6467
Validation	0.9116	0.2775	0.9116	0.5242

#### DeepLab V3+ with backbones efficientnetv2 and SE module

**Fig. 45:**
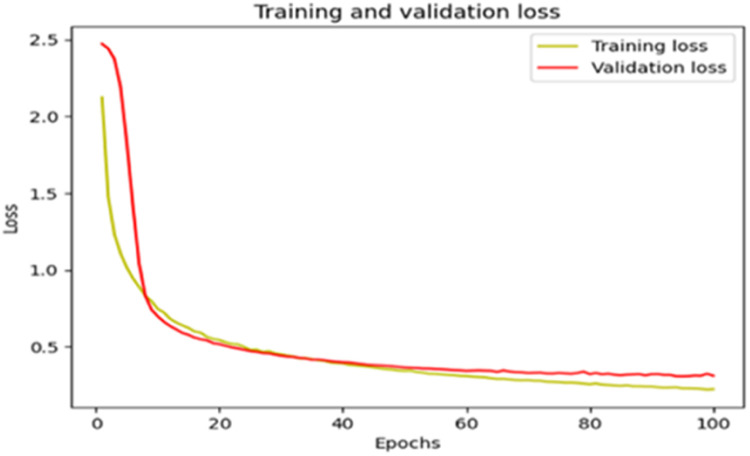
Training and validation” loss.

**Fig. 46:**
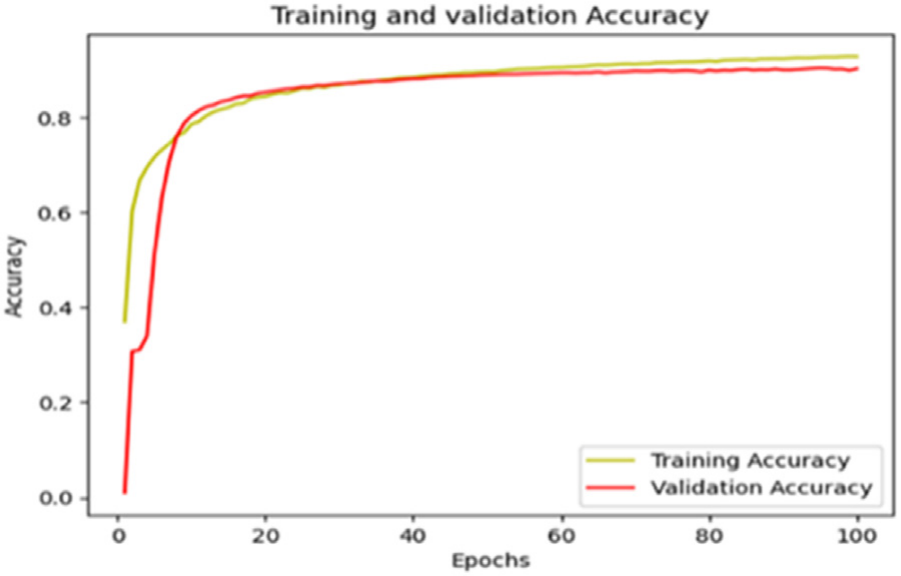
Training and validation” accuracy.

The “Training and validation” loss for the DeepLab V3+ model with the backbone of EfficientNetv2 architecture is shown in Fig. 45 Training and validation” loss. The “Training and validation” Accuracy for the DeepLab V3+ model with the backbone of EfficientNetv2 architecture is indicated in Fig. 46 Training and Validation” Accuracy

**Fig. 47:**
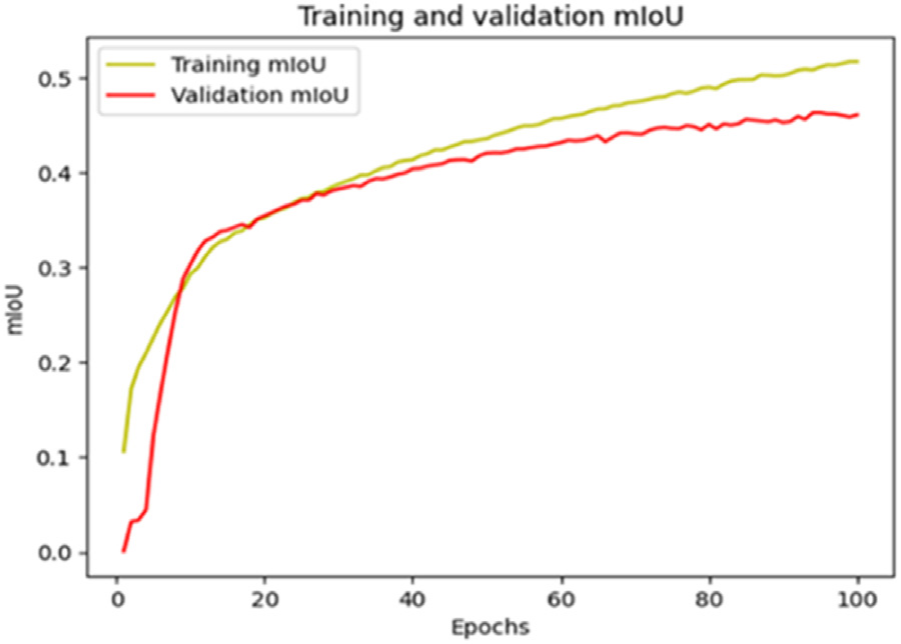
Training and validation” mIoU.

**Fig. 48:**
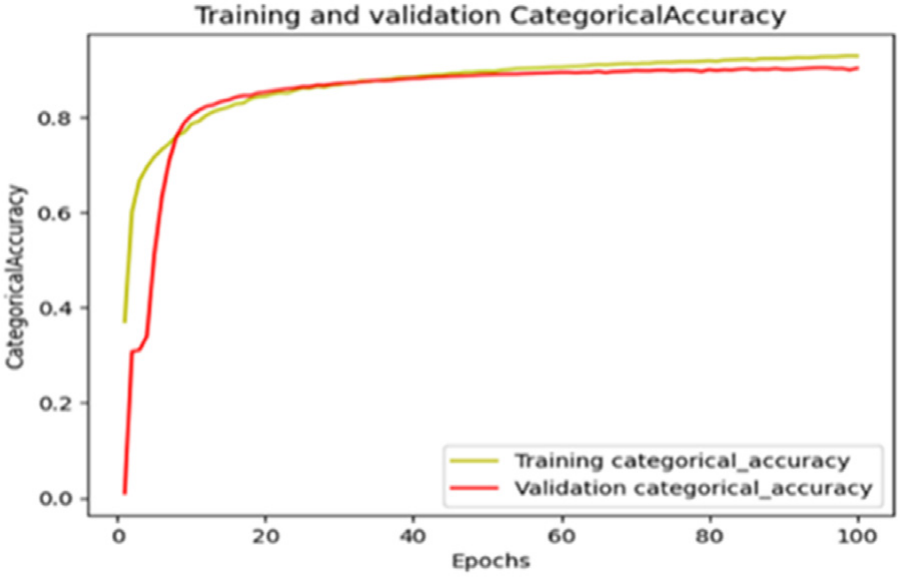
Training and validation” categorical accuracy.

The “Training and validation” mIoU for the DeepLab V3+ model with the backbone of EfficientNetv2 architecture is indicated in Fig. 47. The “Training and validation” Categorical Accuracy is indicated in Fig. 48.

**Fig. 49:**
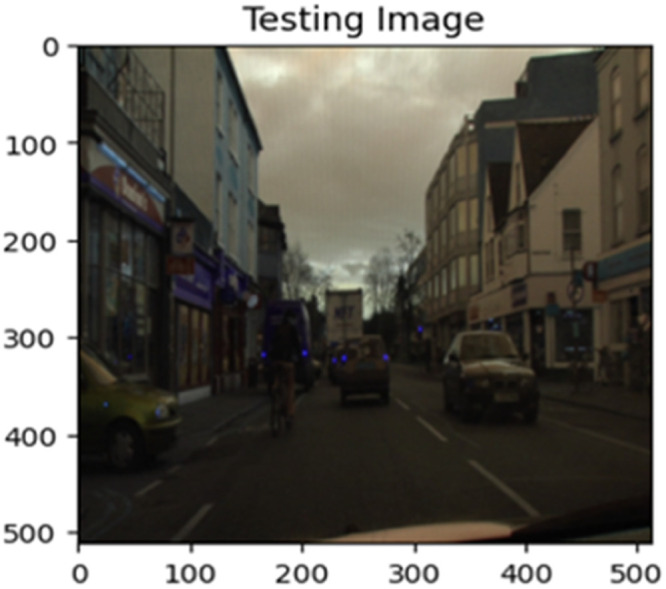
Testing image.

**Fig. 50:**
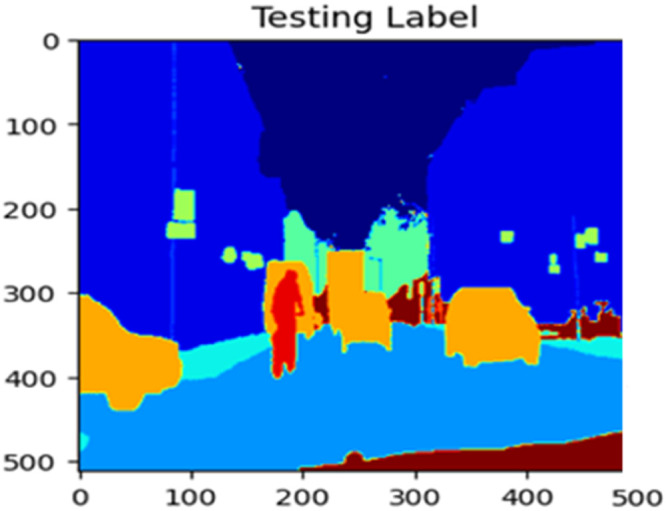
Testing Label.

**Fig. 51:**
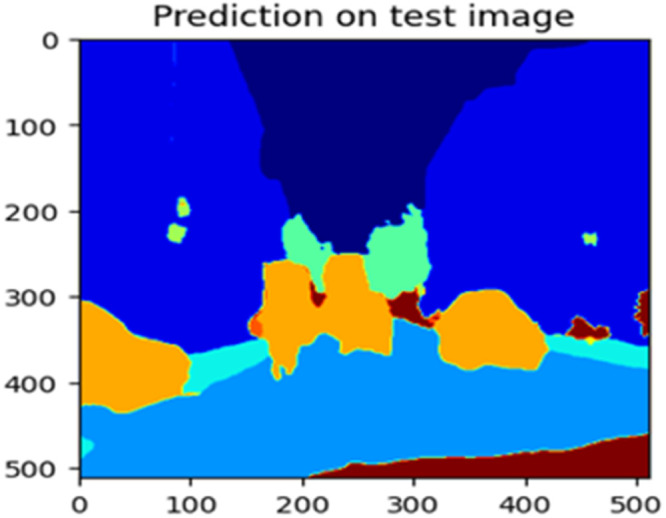
Prediction on test image.

The “testing image”, “testing label”, and prediction of the “test image” for the DeepLab V3+ model with the backbone of EfficientNet with Squeeze Excitation module architecture is indicated in Fig. 49, Fig. 50, Fig. 51 correspondingly.

The summary for the DeepLab V3+ model with the backbone of EfficientNetv2 architecture is shown in Table 12.

**Table 12:** Summary of metrics after 100 Epochs.

Phase	Accuracy	Loss	Categorical Accuracy	Mean IoU
Training	0.9295	0.2199	0.9295	0.5202
Validation	0.9023	0.3119	0.9023	0.4609

#### DeepLab V3+ with backbones efficientnetv2 and CBAM module

**Fig. 52:**
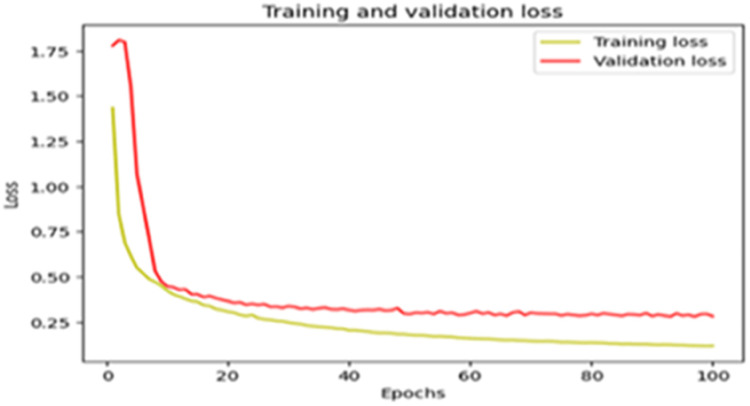
“Training and validation” loss.

**Fig. 53:**
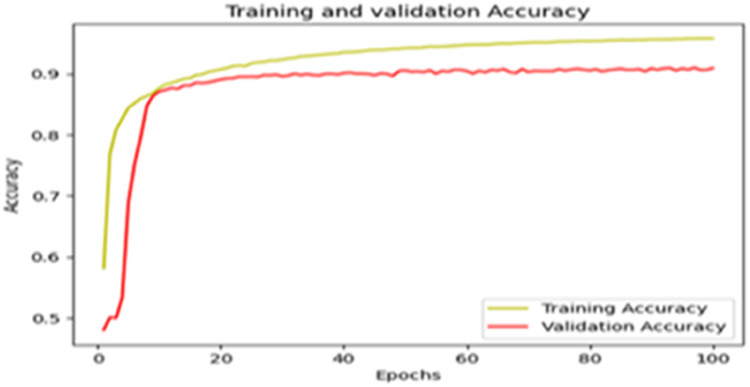
“Training and Validation” Accuracy.

The “Training and validation” loss for the DeepLab V3+ model with the backbone of EfficientNetv2 architecture is indicated in Fig. 52. The “Training and validation” Accuracy for the “DeepLab V3+” model with the backbone of EfficientNetv2 architecture is shown in Fig. 53.

**Fig. 54:**
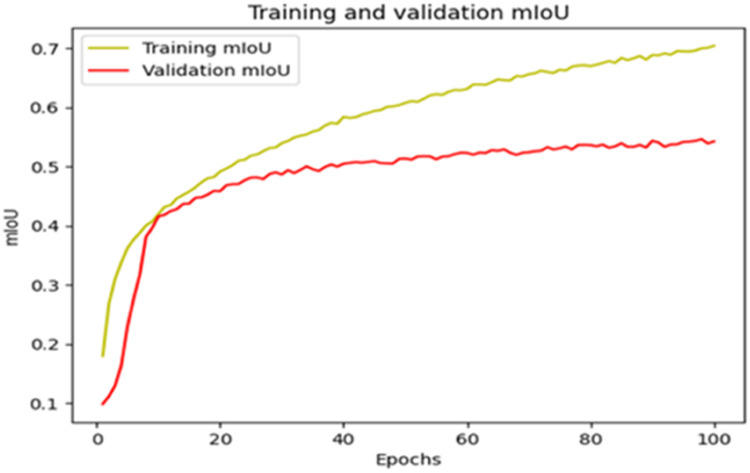
“Training and validation” mIoU.

**Fig. 55:**
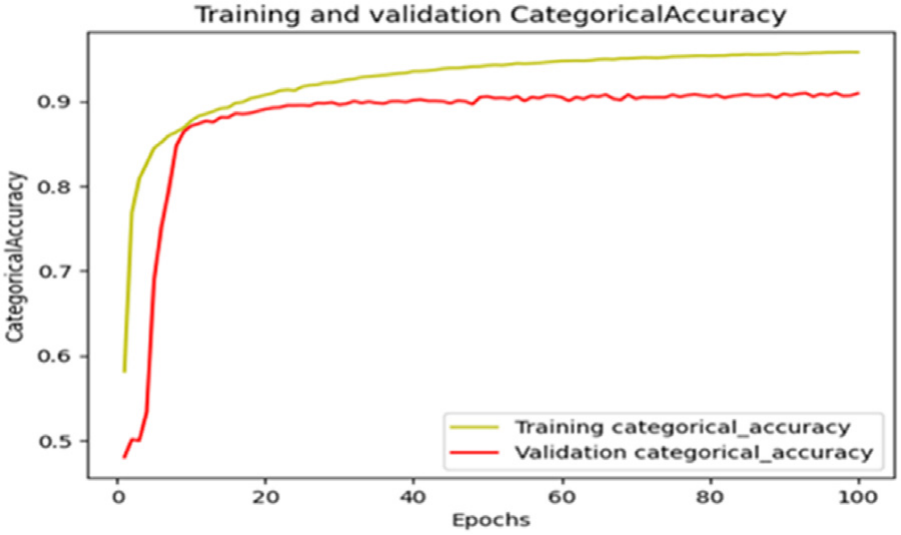
“Training and validation” categorical accuracy.

The “Training and validation” mIoU for the DeepLab V3+ model with the backbone of EfficientNetv2 architecture is indicated in Fig. 54. The “Training and validation” Categorical Accuracy is indicated in Fig. 55.

**Fig. 56:**
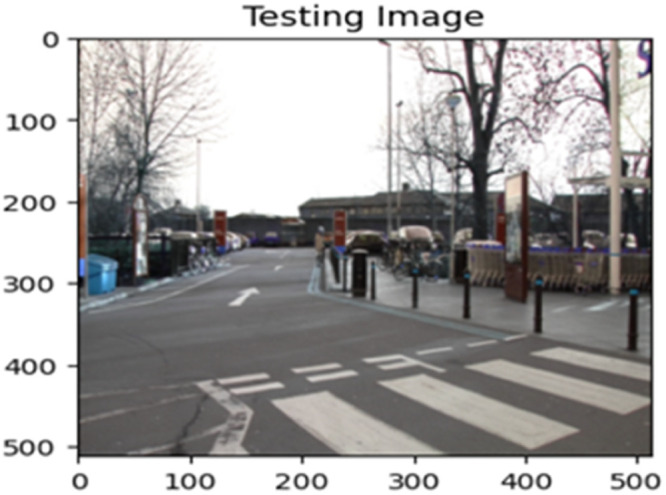
Testing image.

**Fig. 57:**
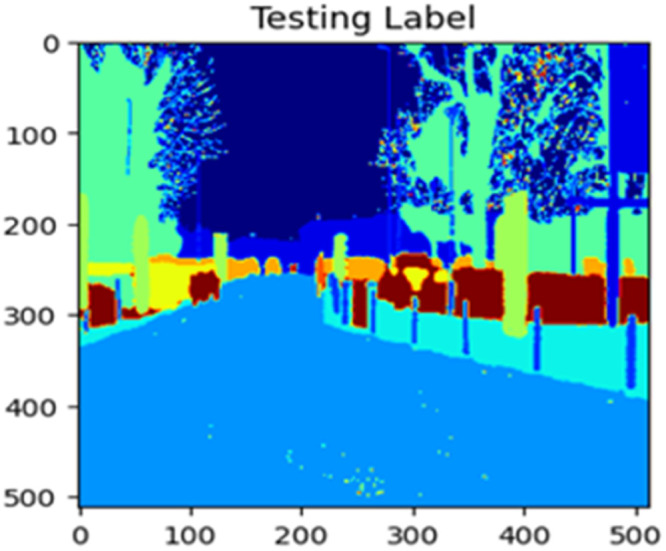
Testing label.

**Fig. 58:**
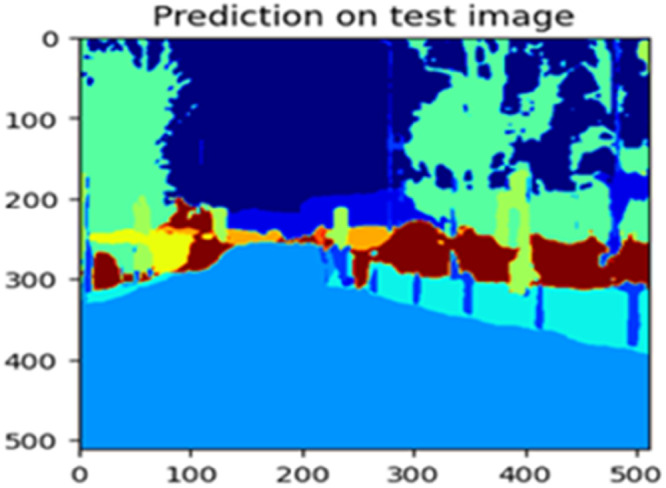
Prediction on test image.

The “testing image”, “testing label”, and prediction of the “test image” for the DeepLab V3+ model with the backbone of EfficientNet with Squeeze Excitation module architecture is indicated in Fig. 56, Fig. 57, Fig. 58 correspondingly. with the backbone of EfficientNetv2 architecture shown in Table 13

**Table 13:** Summary of metrics after 100 Epochs.

Phase	Accuracy	Loss	Categorical Accuracy	Mean IoU
Training	0.9592	0.1156	0.9592	0.7003
Validation	0.9094	0.2825	0.9094	0.5423

## Limitations

Results are based on smaller samples data set and the limitation of the proposed work is the time complexity of limited iterations of the algorithm due to limited resources. Increase in number of classes for semantic segmentation may also have limitation.

## Ethics statements

“All authors declare that this work complies with ethical guidelines set by MethodsX”

## CRediT authorship contribution statement

**Javed Subhedar:** Conceptualization, Methodology, Writing – original draft. **Mrinal R Bachute:** Writing – review & editing, Visualization, Investigation.

## Declaration of competing interest

“The authors declare that they have no known competing financial interests or personal relationships that could have appeared to influence the work reported in this paper.”

## Data Availability

Data will be made available on request.

## References

[1] E.L. Chao and M. Kratsios, “Ensuring American leadership in automated vehicle technologies automated vehicles 4.0,” 2020.

[2] Bachute M.R., Subhedar J.M. (Dec. 2021). Autonomous driving architectures: insights of machine learning and deep learning algorithms. Mach. Learn. Appl..

[3] Feng D. (2021). Deep multi-modal object detection and semantic segmentation for autonomous driving: datasets, methods, and challenges. IEEE Trans. Intell. Transp. Syst..

[4] S.A.E. International, “Taxonomy and definitions for terms related to driving automation systems for on-road motor vehicles J3016,” 2018. 10.4271/J3016_201806.

[5] NHTSA, “Automated driving systems 2.0: a vision for safety,” 2017. [Online]. Available: https://www.nhtsa.gov/sites/nhtsa.dot.gov/files/documents/13069a-ads2.0_090617_v9a_tag.pdf.

[6] C. Liu et al., “Auto-DeepLab: hierarchical neural architecture search for semantic image segmentation,” Jan. 2019, [Online]. Available: http://arxiv.org/abs/1901.02985.

[7] H. Zhao, J. Shi, X. Qi, X. Wang, and J. Jia, “Pyramid scene parsing network,” 2017. 10.1109/CVPR.2017.660.

[8] V. Badrinarayanan, A. Kendall, and R. Cipolla, “SegNet: a deep convolutional encoder-decoder architecture for image segmentation,” 2017. 10.1109/TPAMI.2016.2644615. 10.1109/TPAMI.2016.2644615 28060704

[9] Ronneberger O., Fischer P., Brox T. (2015). U-Net: convolutional networks for biomedical image segmentation. Lect. Notes Comput. Sci..

[10] Subhedar J., Bachute M.R., Kotecha K. (2024). Optimisation of semantic segmentation algorithm for autonomous driving using U-NET architecture. IAES Int. J. Artif. Intell. (IJ-AI).

[11] L.C. Chen, G. Papandreou, I. Kokkinos, K. Murphy, and A.L. Yuille, “Semantic image segmentation with deep convolutional nets and fully connected CRFs,” 2014, [Online]. Available: http://arxiv.org/abs/1412.7062. 10.1109/TPAMI.2017.2699184 28463186

[12] L.C. Chen, G. Papandreou, F. Schroff, and H. Adam, “Rethinking atrous convolution for semantic image segmentation,” 2017, [Online]. Available: http://arxiv.org/abs/1706.05587.

[13] L.C. Chen, Y. Zhu, G. Papandreou, F. Schroff, and H. Adam, “Encoder-decoder with atrous separable convolution for semantic image segmentation.” Accessed: Apr. 13, 2024. [Online]. Available: https://arxiv.org/pdf/1802.02611.

[14] Hsu C.Y., Hu R., Xiang Y., Long X., Li Z. (2022). Improving the Deeplabv3+ model with attention mechanisms applied to eye detection and segmentation. Mathematics.

[15] Zhang C., Chen X., Ji S. (2022). Semantic image segmentation for sea ice parameters recognition using deep convolutional neural networks. Int. J. Appl. Earth Obs. Geoinf..

[16] J. Li et al., “Lane-DeepLab: lane semantic segmentation in automatic driving scenarios for high-definition maps”, 10.1016/j.neucom.2021.08.105.

[17] Wang J., Liu X. (2021). Medical image recognition and segmentation of pathological slices of gastric cancer based on Deeplab v3 + neural network. Comput. Methods Programs Biomed..

[18] Wang Y. (2021). Mask DeepLab: end-to-end image segmentation for change detection in high-resolution remote sensing images. Int. J. Appl. Earth Obs. Geoinf..

[19] J. Czajkowska, P. Badura, S. Korzekwa, and A. Płatkowska-Szczerek, “Automated segmentation of epidermis in high-frequency ultrasound of pathological skin using a cascade of DeepLab v3+ networks and fuzzy connectedness,” 2021, 10.1016/j.compmedimag.2021.102023. 10.1016/j.compmedimag.2021.102023 34883364

[20] Zhou Z., Zheng Y., Zhang J., Yang H. (2023). Fast detection algorithm for cracks on tunnel linings based on deep semantic segmentation. Front. Struct. Civ. Eng..

[21] V. Zamani, H. Taghaddos, Y. Gholipour, and H. Pourreza, “Deep semantic segmentation for visual scene understanding of soil types,” 2022, 10.1016/j.autcon.2022.104342.

[22] Wu D., Yin X., Jiang B., Jiang M., Li Z., Song H. (2020). Detection of the respiratory rate of standing cows by combining the Deeplab V3+ semantic segmentation model with the phase-based video magnification algorithm. Biosyst. Eng..

[23] Kong Y., Liu Y., Yan B., Leung H., Peng X. (2021). A novel deeplabv3+ network for sar imagery semantic segmentation based on the potential energy loss function of gibbs distribution. Remote Sens..

[24] Zeng H., Peng S., Li D. (2020). Deeplabv3+ semantic segmentation model based on feature cross attention mechanism. J. Phys. Conf. Ser..

[25] University Of Cambridge, “CAMVid data set .” accessed: may 16, 2024. [Online]. Available: https://www.kaggle.com/datasets/carlolepelaars/camvid?resource=download.

[26] “Semantic segmentation using KerasCV DeepLabv3+.2025 Accessed: May 28, 2024. [Online]. Available: https://github.com/spmallick/learnopencv/tree/master/Semantic-Segmentation-using-KerasCV-with-DeepLabv3-Plus.

[27] J. Hu, L. Shen, S. Albanie, G. Sun, and E. Wu, “Squeeze-and-excitation networks,” 2017, [Online]. Available: http://arxiv.org/abs/1709.01507. 10.1109/TPAMI.2019.2913372 31034408

[28] S. Woo, J. Park, J.Y. Lee, and I.S. Kweon, “CBAM: convolutional block attention module,” 2018, [Online]. Available: http://arxiv.org/abs/1807.06521.

[29] Chen H., Qin Y., Liu X., Wang H., Zhao J. (2024). An improved DeepLabv3+ lightweight network for remote-sensing image semantic segmentation. Complex Intell. Syst..

[30] “Category Accuracy.” Accessed: may 22, 2024. [Online]. Available: 2025 https://keras.io/api/metrics/accuracy_metrics/.

